# Supplementary material for: The phytochrome interacting proteins ERF55 and ERF58 repress light-induced seed germination in Arabidopsis thaliana
Source: Nat Commun. 2022 Mar 29;13:1656. doi: 10.1038/s41467-022-29315-3 (PMC8964797; doi:10.1038/s41467-022-29315-3)
Supplement: Supplementary file 1 — Supplementary Information [file 41467_2022_29315_MOESM1_ESM.pdf]

SUPPLEMENTARY INFORMATION

The phytochrome interacting proteins ERF55 and ERF58 repress light-induced seed germination in *Arabidopsis thaliana*

Zenglin Li[1], David J. Sheerin[1], Edda von Roepenack-Lahaye[2], Mark Stahl[2], and Andreas Hiltbrunner[1,3,*]

[1] Institute of Biology II, Faculty of Biology, University of Freiburg, Freiburg, Germany

[2] Centre for Plant Molecular Biology, University of Tübingen, Tübingen, Germany

[3] Signalling Research Centres BIOSS and CIBSS, University of Freiburg, Freiburg, Germany

[*] Corresponding author; andreas.hiltbrunner@biologie.uni-freiburg.de

SUPPLEMENTARY FIGURES

a

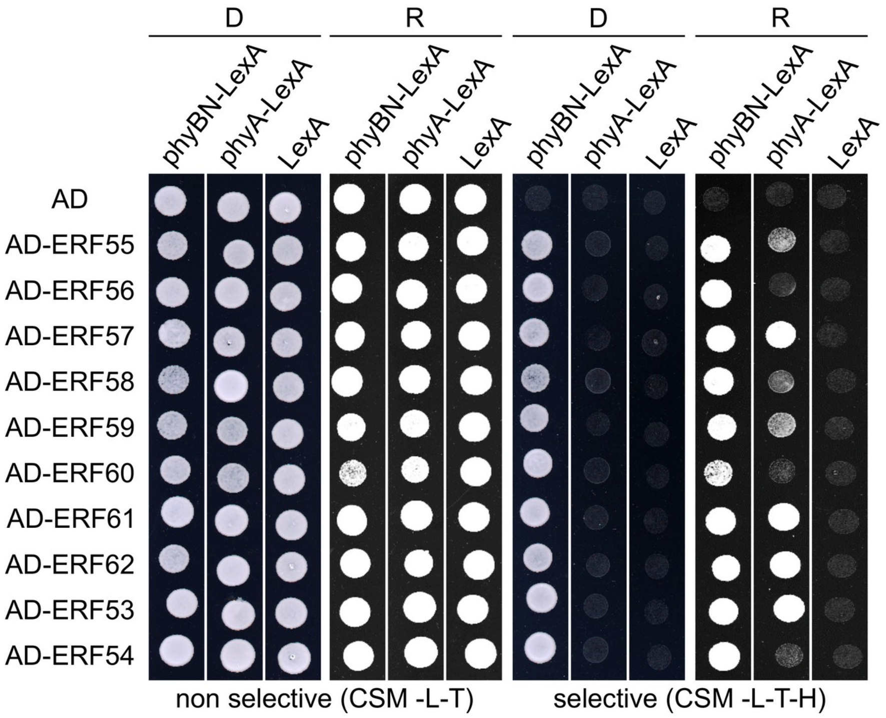

b

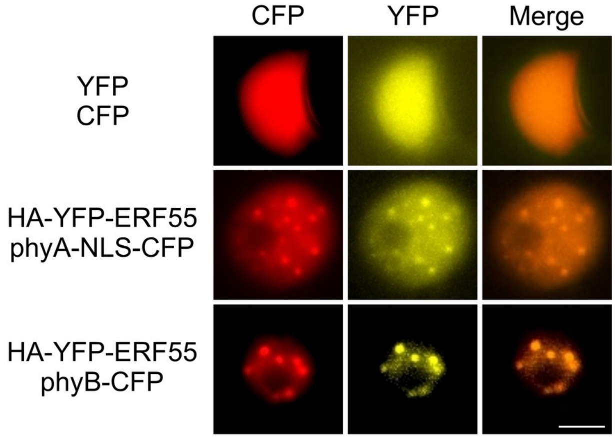

Supplementary Fig. 1 Group A6 ERFs interact with phyA and phyB. **a** Y2H growth assay. phyA- or the N-terminal half of phyB (phyBN) fused to LexA and ERFs from group A6 of the AP2/ERF transcription factor family fused to the GAL4 AD were expressed in yeast. Yeast cells were grown on CSM -L-T plates or CSM -L-T-H plates supplemented with PCB. Plates were incubated in the dark (D) or R light (R). **b** Co-localisation of ERF55 with phyA and phyB in tobacco. *p35S:HA-YFP-ERF55* and either *p35S:PHYA-NLS-CFP* or *p35S:PHYB-CFP* were transiently co-expressed in tobacco leaf epidermis cells by agro infiltration; empty *p35S:YFP* and *p35S:CFP* vectors were used as control. YFP and CFP signals were detected by epifluorescence microscopy. The YFP/CFP control is also shown in **Fig. 1c**. Scale bar represents 5 µm. Experiments were repeated three times with similar results.

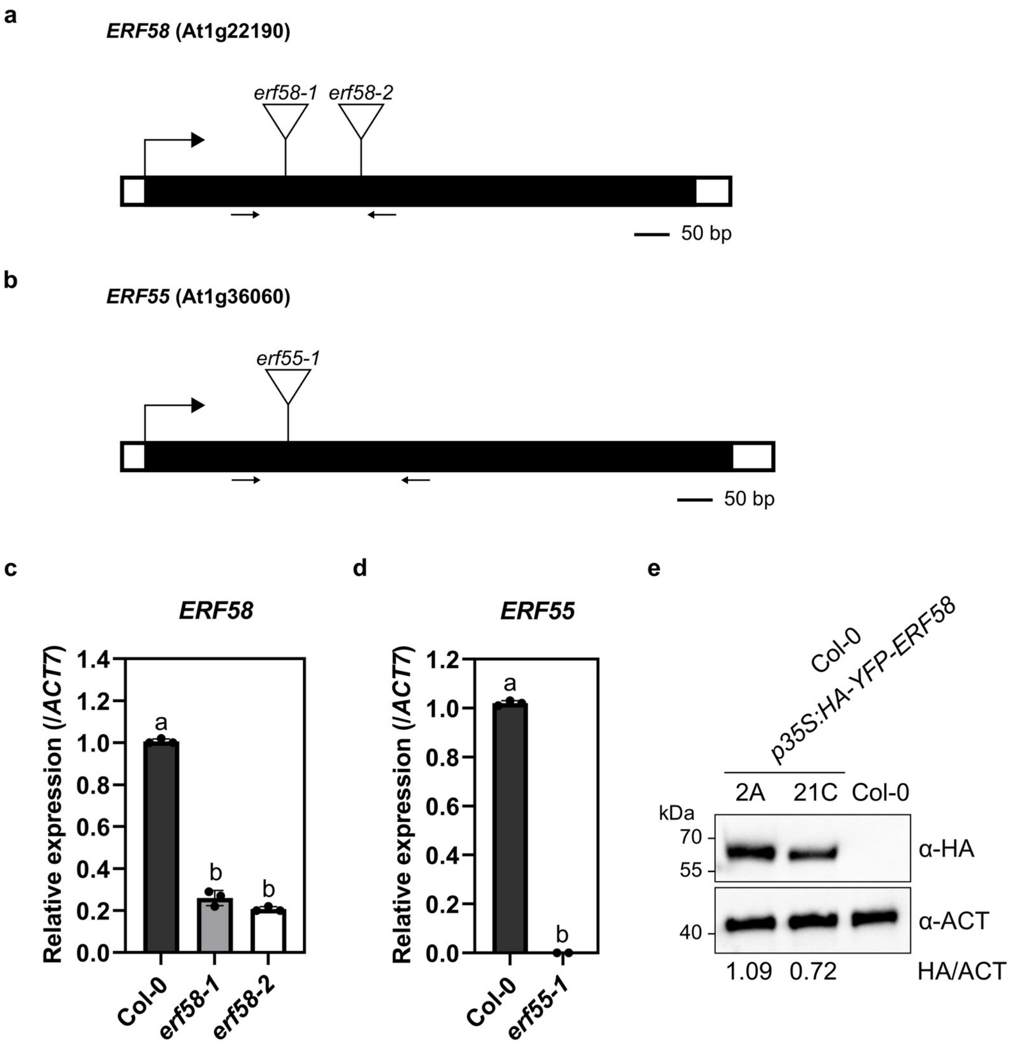

Supplementary Fig. 2 *ERF58* and *ERF55* T-DNA insertion and ERF58 overexpression lines. **a, b** Diagrams showing the position of the T-DNA insertions in the *ERF58* locus in *erf58-1* and *erf58-2* **(a)**, and in the *ERF55* locus in *erf55-1* **(b)**. Black box, exon; white boxes, UTRs; arrow above boxes, transcription start site; arrows below boxes, primers used for RT-qPCR in **c, d**. **c, d** RT-qPCR to detect *ERF58* **(c)** and *ERF55* **(d)** transcript levels in 4 day old dark-grown wild-type and *erf58-1*, *erf58-2* **(c)** or *erf55-1* **(d)** mutant seedlings. *ACT7* was used as an internal control. Values are means of three replicates ±SD. Different letters indicate significant differences as determined by one-way ANOVA followed by post-hoc Tukey's HSD test; $p<0.05$. **e** HA-YFP-ERF58 protein levels in two independent ERF58ox lines (2A and 21C). Seeds were germinated for 12 hours in the dark. Total protein was extracted and analysed by SDS-PAGE and immunoblotting with α-HA; α-ACT was used to detect ACTIN as loading control. Col-0 was included as negative control. Signals detected by α-HA and α-ACT were quantified using ImageJ; numbers below the membrane show the HA/ACT ratio. The experiment was repeated thee times with similar results.

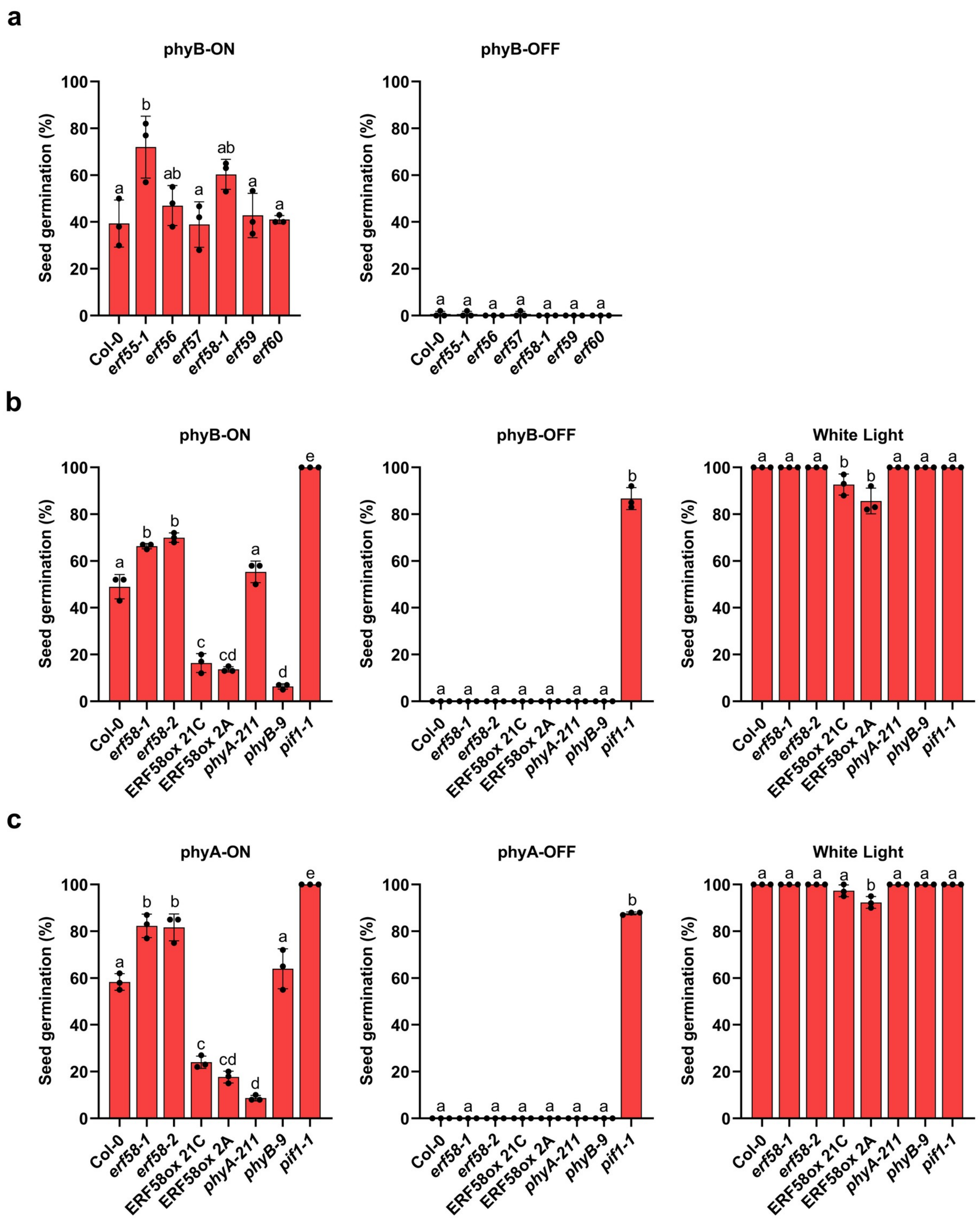

Supplementary Fig. 3 ERF55 and ERF58 are negative regulators of the completion of seed germination. **a-c** Final cumulative seed germination of different genotypes was quantified under phyA- **(c)** or phyB-specific **(a, b)** conditions. Light treatments (phyA-ON/OFF, phyB-ON/OFF) are described in **Fig. 2a, b**. Bars represent mean cumulative germination percentages of three replicates ±SD. Different letters indicate significant differences as determined by one-way ANOVA followed by post-hoc Tukey's HSD test; $p<0.05$.

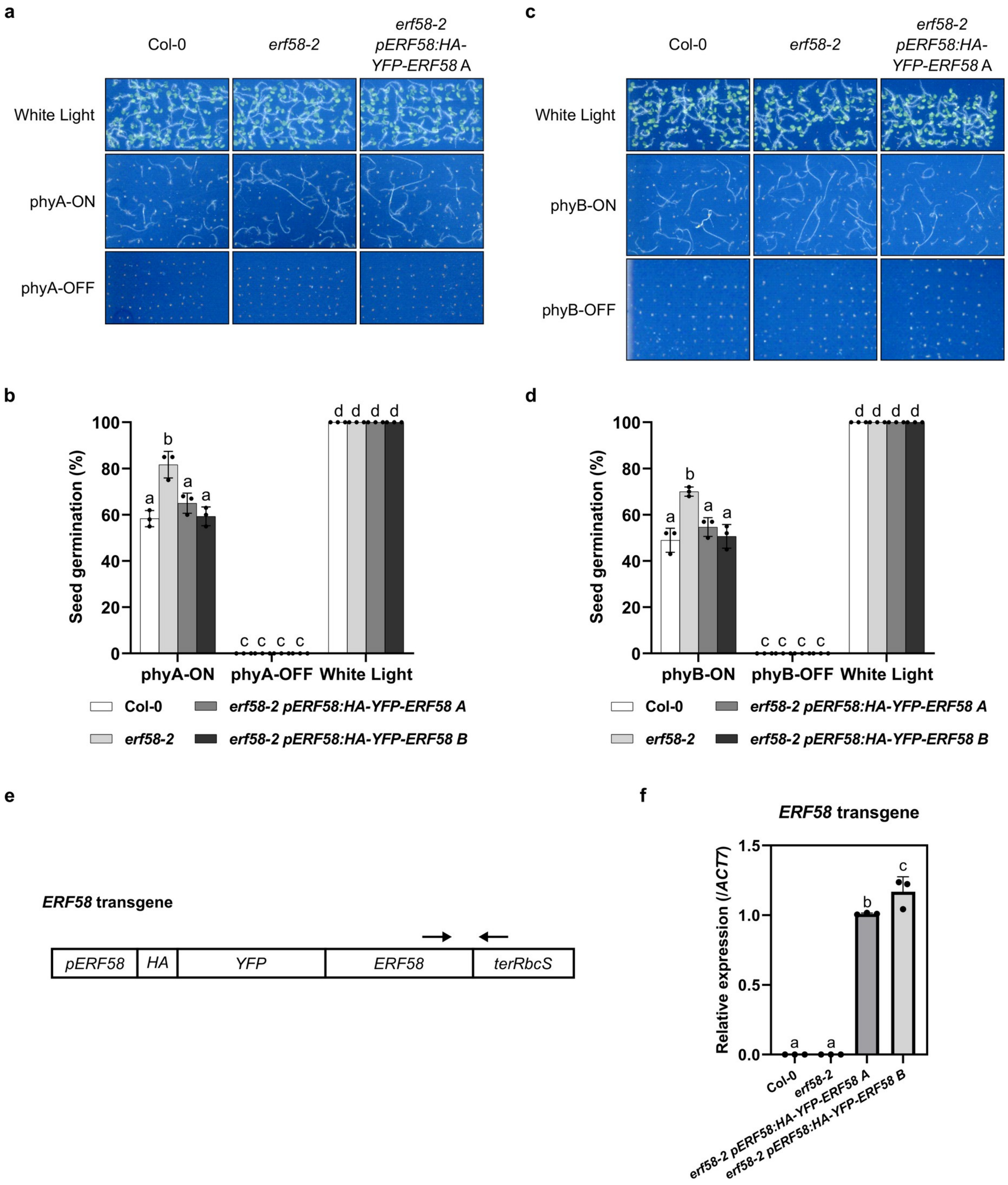

Supplementary Fig. 4 Complementation of the *erf58-2* mutant by expression of *pERF58:HA-YFP-ERF58*. **a-d** Test of completion of germination for wild-type (Col-0), *erf58-2*, and two independent *erf58-2 pERF58:HA-YFP-ERF58* lines (A, B) under phyA-ON/OFF **(a, b)** or phyB-ON/OFF **(c, d)** conditions. Light treatments (phyA-ON/OFF, phyB-ON/OFF) are described in **Fig. 2a, b**. **b, d** Bars show mean cumulative germination percentages of three replicates ±SD. **e** Schematic representation of the *pERF58:HA-YFP-ERF58* transgene. Arrows indicate primers used for RT-qPCR in **f**. **f** RT-qPCR analysis of transgene expression

in complementing lines. Total RNA was extracted from seeds germinating for 12 hours in the dark and used for RT-qPCR with transgene-specific primers. *ACT7* was used as an internal control. Values are means of three replicates ±SD. **b, d, f** Different letters indicate significant differences as determined by one-way ANOVA followed by post-hoc Tukey's HSD test; $p<0.05$.

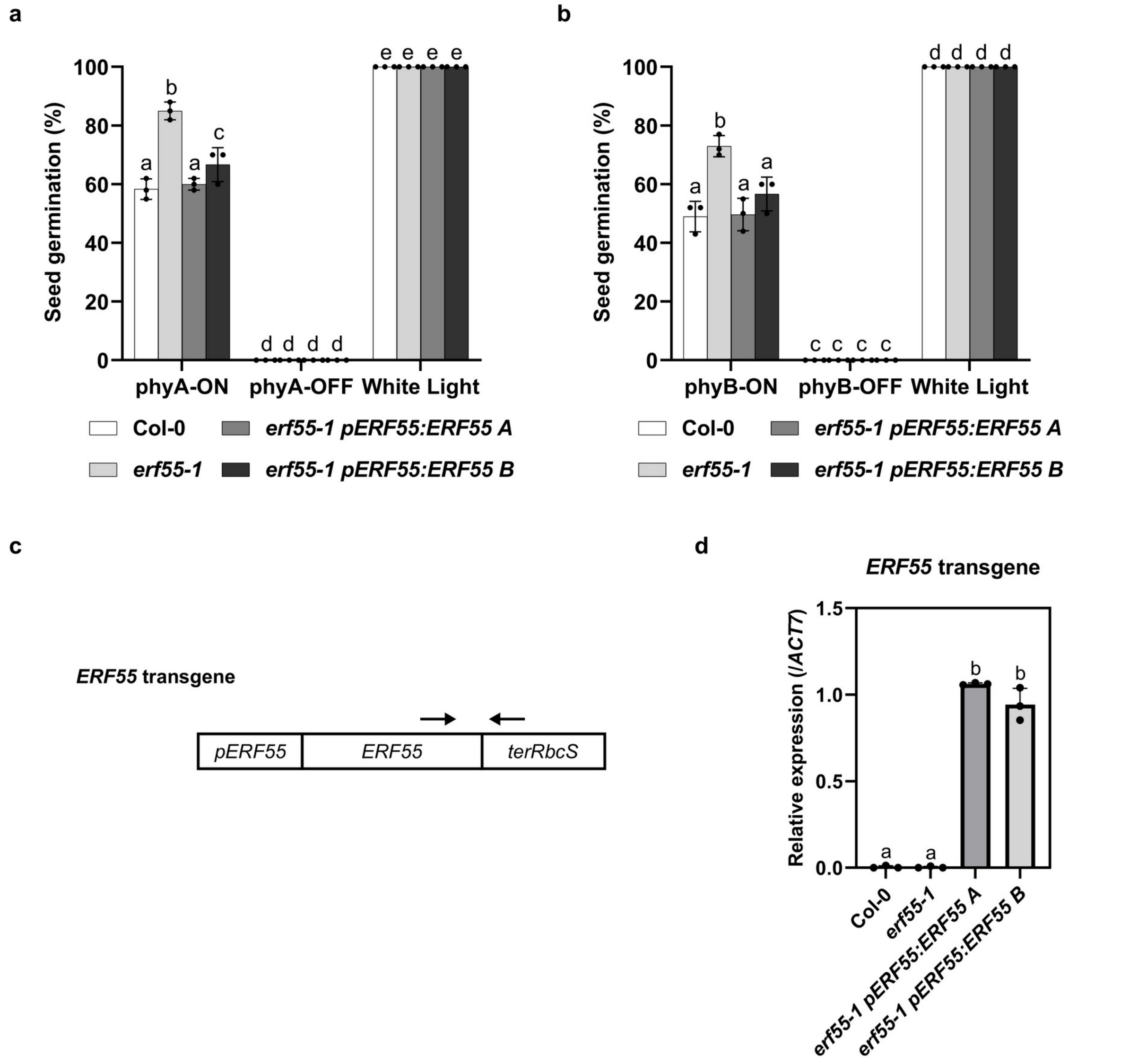

Supplementary Fig. 5 Complementation of the *erf55-1* mutant by expression of *pERF55:ERF55*. **a-b** Test of completion of germination for wild-type (Col-0), *erf55-1*, and two independent *erf55-1 pERF55:ERF55* lines (A, B) under phyA-ON/OFF **(a)** or phyB-ON/OFF **(b)** conditions. Light treatments (phyA-ON/OFF, phyB-ON/OFF) are described in **Fig. 2a, b**. Bars show mean cumulative germination percentages of three replicates ±SD. **c** Schematic representation of the *pERF55:ERF55* transgene. Arrows indicate primers used for RT-qPCR in **d**. **d** RT-qPCR analysis of transgene expression in complementing lines. Total RNA was extracted from seeds germinating for 12 hours in the dark and used for RT-qPCR with transgene-specific primers. *ACT7* was used as an internal control. Values are means of three replicates ±SD. **a, b, d** Different letters indicate significant differences as determined by one-way ANOVA followed by post-hoc Tukey's HSD test; $p<0.05$.

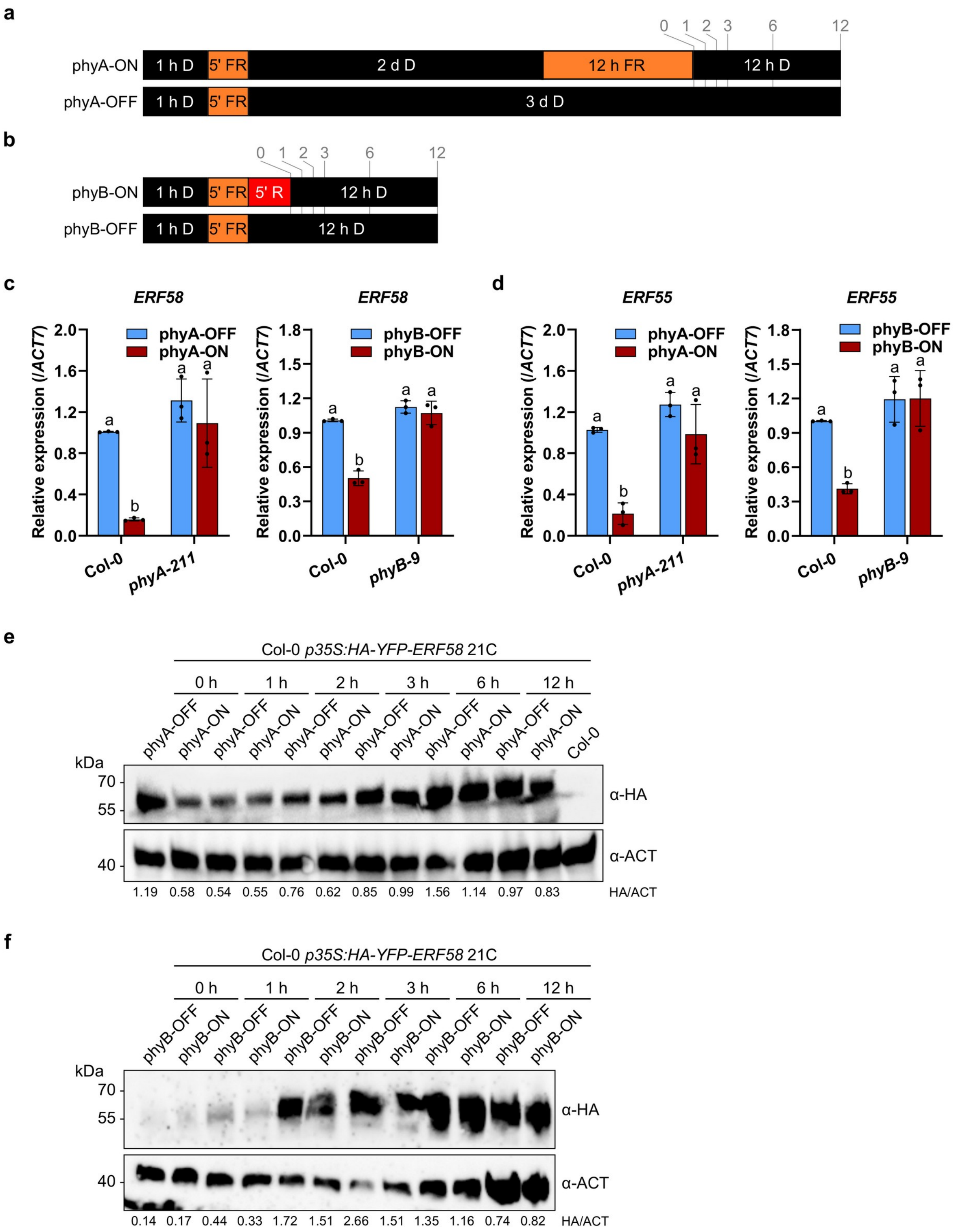

Supplementary Fig. 6 PhyA and phyB repress *ERF55* and *ERF58* expression. **a, b** Light treatments used to test for phyA- and phyB-dependent regulation of *ERF55* and *ERF58* expression in germinating seeds. Seeds were imbibed for 1 hour in the dark (D), treated for 5 min with FR light and then exposed to light conditions that specifically activate phyA (phyA-ON: 2 days in D → 12 hours in FR light → 12 hours in D) or phyB (phyB-ON: 5 min in R light

→ 12 hours in D). phyA and phyB are inactive under phyA-OFF and phyB-OFF conditions. Time points for harvesting samples for testing HA-YFP-ERF58 protein stability **(e, f)** are indicated in grey. **c, d** RT-qPCR analysis of *ERF58* **(c)** and *ERF55* expression **(d)** in seeds germinating under phyA-ON/OFF or phyB-ON/OFF conditions. *ACT7* was used as an internal control. Values are means of three replicates ±SD. Different letters indicate significant differences as determined by two-way ANOVA followed by post-hoc Tukey's HSD test; $p<0.05$. **e, f** HA-YFP-ERF58 protein stability. Col-0 *p35S:HA-YFP-ERF58* seeds (line 21C) were germinated under phyA-ON/OFF **(e)** or phyB-ON/OFF **(f)** conditions followed by 0, 1, 2, 3, 6, or 12 hours incubation in the dark as indicated in panel **a**. Col-0 was used as negative control. Total protein was extracted and analysed by SDS-PAGE and immunoblotting with α-HA; α-ACT was used to detect ACTIN as loading control. Signals detected by α-HA and α-ACT were quantified using ImageJ; numbers below the membrane show the HA/ACT ratio. Experiments were repeated three times with similar results.

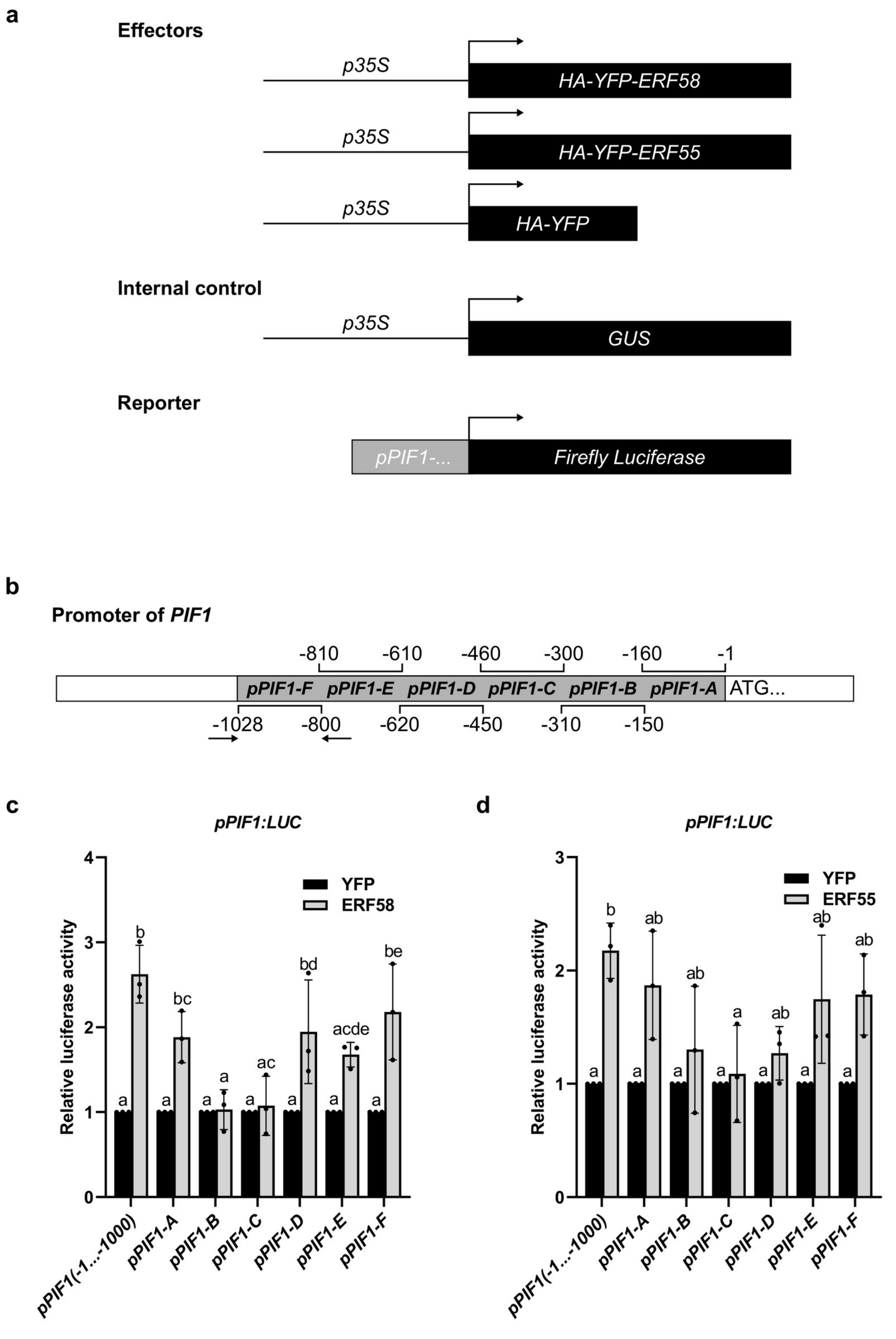

Supplementary Fig. 7 ERF55 and ERF58 activate the *PIF1* promoter. **a** Schematic representation of effector, reporter, and control constructs used for transient transactivation assays in tobacco. Effector and control constructs were driven by the *35S* promoter; *PIF1* promoter fragments were used to drive expression of firefly luciferase. *GUS* driven by the *35S* promoter was used as an internal control. **b** Diagram depicting the *PIF1* promoter and

promoter fragments (*pPIF1-A*, *pPIF1-B*, *pPIF1-C*, *pPIF1-D*, *pPIF1-E*, *pPIF1-F*) used in the transactivation assay. Arrows indicate primers used for ChIP-qPCR and for PCR amplification of probes for EMSAs **(Fig. 3f, g; Fig. 4c, d, e, g)**. **c, d** Transactivation assay in tobacco. The effector constructs *p35S:HA-YFP-ERF58* **(c)** or *p35S:HA-YFP-ERF55* **(d)**, reporter constructs (*pPIF1* promoter fragments driving expression of *LUC*), and *p35S:GUS* (used for normalisation) were transiently co-expressed in tobacco leaf epidermis cells by agro infiltration. LUC and GUS activity was measured in protein extracts from infiltrated leaves. Bars show mean relative LUC activity (LUC activity divided by GUS activity) of three replicates ±SD. Different letters indicate significant differences as determined by two-way ANOVA followed by post-hoc Tukey's HSD test; $p<0.05$.

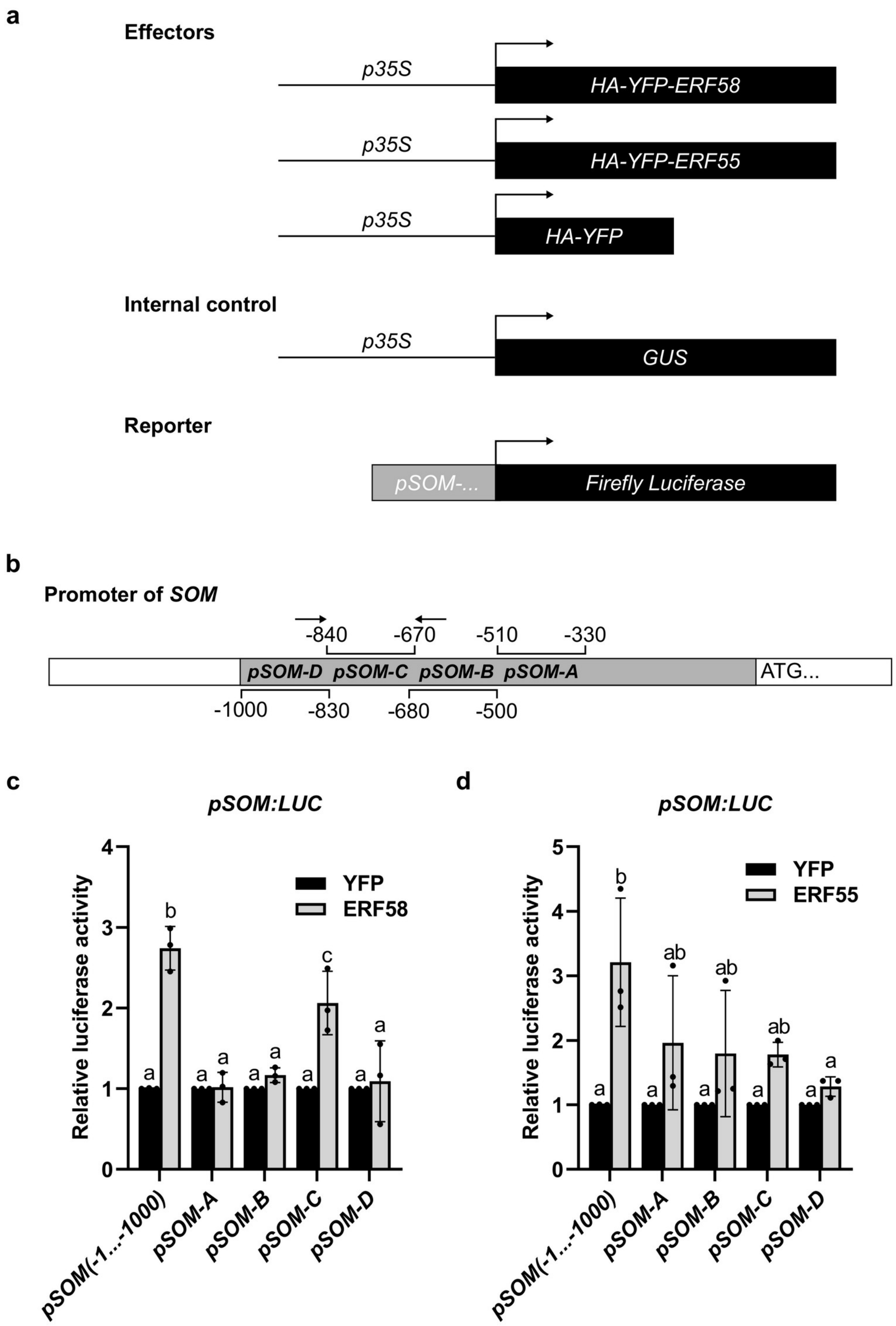

Supplementary Fig. 8 ERF55 and ERF58 activate the *SOM* promoter. **a** Schematic representation of effector, reporter, and control constructs used for transient transactivation assays in tobacco. Effector and control constructs were driven by the *35S* promoter; *SOM* promoter fragments were used to drive expression of firefly luciferase. *GUS* driven by the *35S* promoter was used as an internal control. **b** Diagram depicting the *SOM* promoter and promoter fragments (*pSOM-A*, *pSOM-B*, *pSOM-C*, *pSOM-D*) used in the transactivation

assay. Arrows indicate primers used for ChIP-qPCR and for PCR amplification of probes for EMSAs **(Fig. 3f, g; Fig. 4c, d, f, h)**. **c, d** Transactivation assay in tobacco. The effector constructs *p35S:HA-YFP-ERF58* **(c)** or *p35S:HA-YFP-ERF55* **(d)**, reporter constructs (*pSOM* promoter fragments driving expression of *LUC*), and *p35S:GUS* (used for normalisation) were transiently co-expressed in tobacco leaf epidermis cells by agro infiltration. LUC and GUS activity was measured in protein extracts from infiltrated leaves. Bars show mean relative LUC activity (LUC activity divided by GUS activity) of three replicates ±SD. Different letters indicate significant differences as determined by two-way ANOVA followed by post-hoc Tukey's HSD test; $p<0.05$.

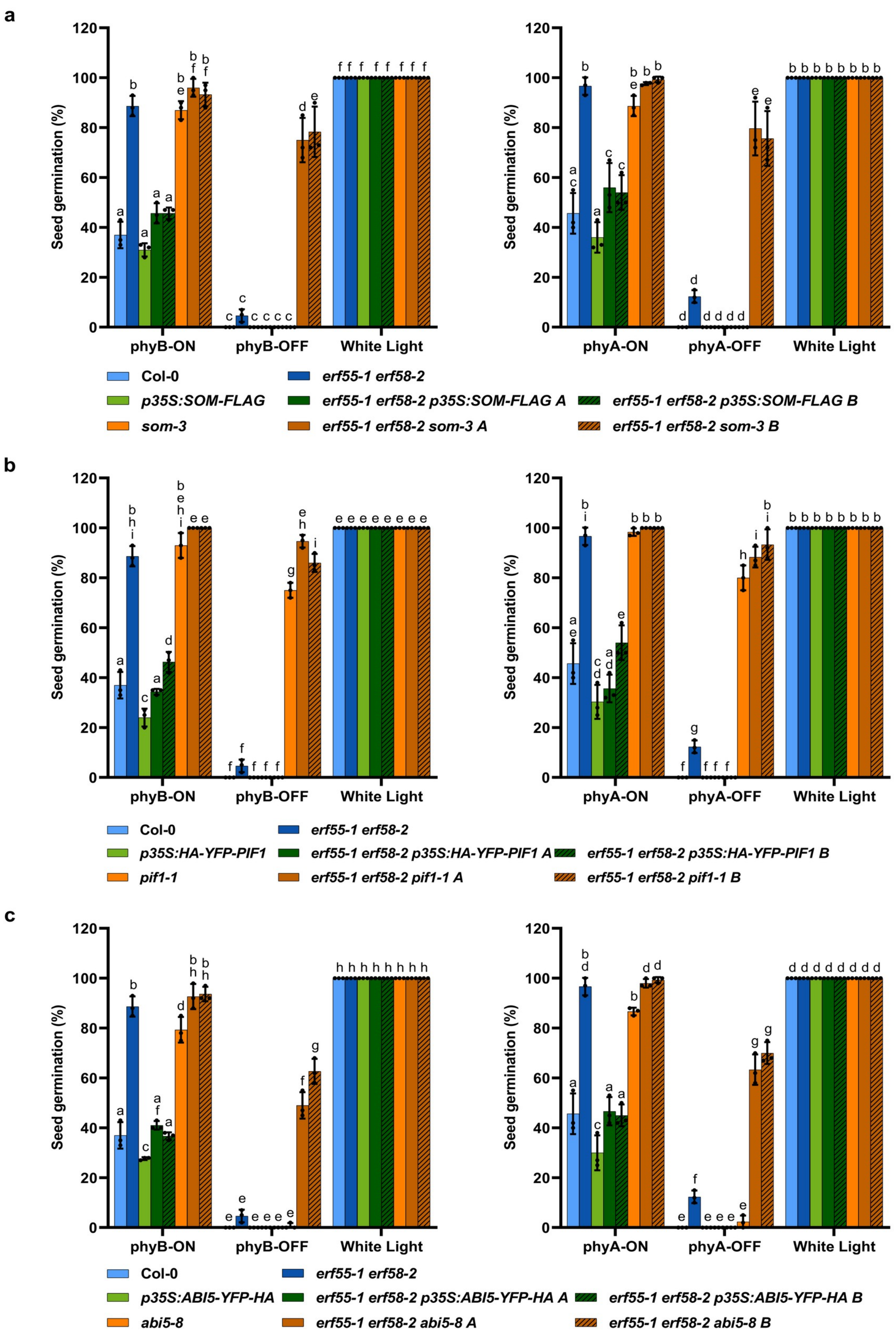

Supplementary Fig. 9 Completion of seed germination of *erf55-1 erf58-2* in *SOM*, *PIF1*, or *ABI5* mutant or overexpression background. **a-c** Completion of seed germination of indicated

genotypes was tested under phyA- and phyB-specific conditions. Light treatments (phyA-ON/OFF, phyB-ON/OFF) are described in **Fig. 2a, b**. The same data set is shown for Col-0 and *erf55-1 erf58-2* in **a-c**. Bars represent mean cumulative germination percentages of three replicates ±SD. Different letters indicate significant differences as determined by two-way ANOVA followed by post-hoc Tukey's HSD test; $p<0.05$.

a

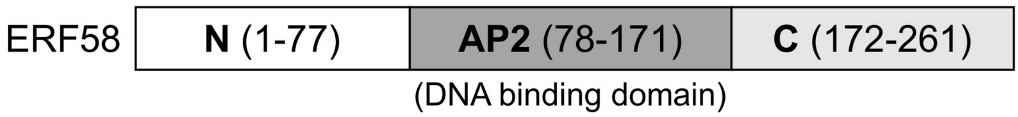

b

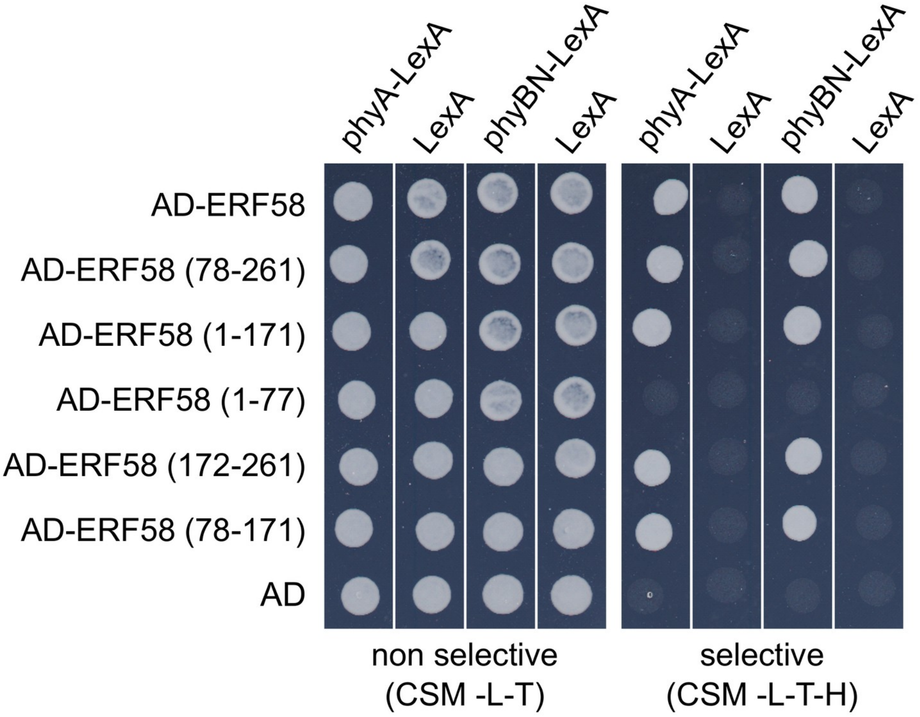

Supplementary Fig. 10 The AP2 domain of ERF58 interacts with phyA and phyB. **a** Diagram depicting the domain structure of ERF58; numbers indicate amino acid positions. **b** Y2H growth assay. Full-length phyA or the N-terminal half of phyB (phyBN) fused to LexA and different ERF58 fragments (numbers indicate amino acid positions) fused to the GAL4 AD were expressed in yeast. Yeast cells were grown on CSM -L-T plates or CSM -L-T-H plates supplemented with PCB. Plates were incubated in 2 μmol m^{-2} s^{-1} R light. The experiment was repeated three times with similar results.

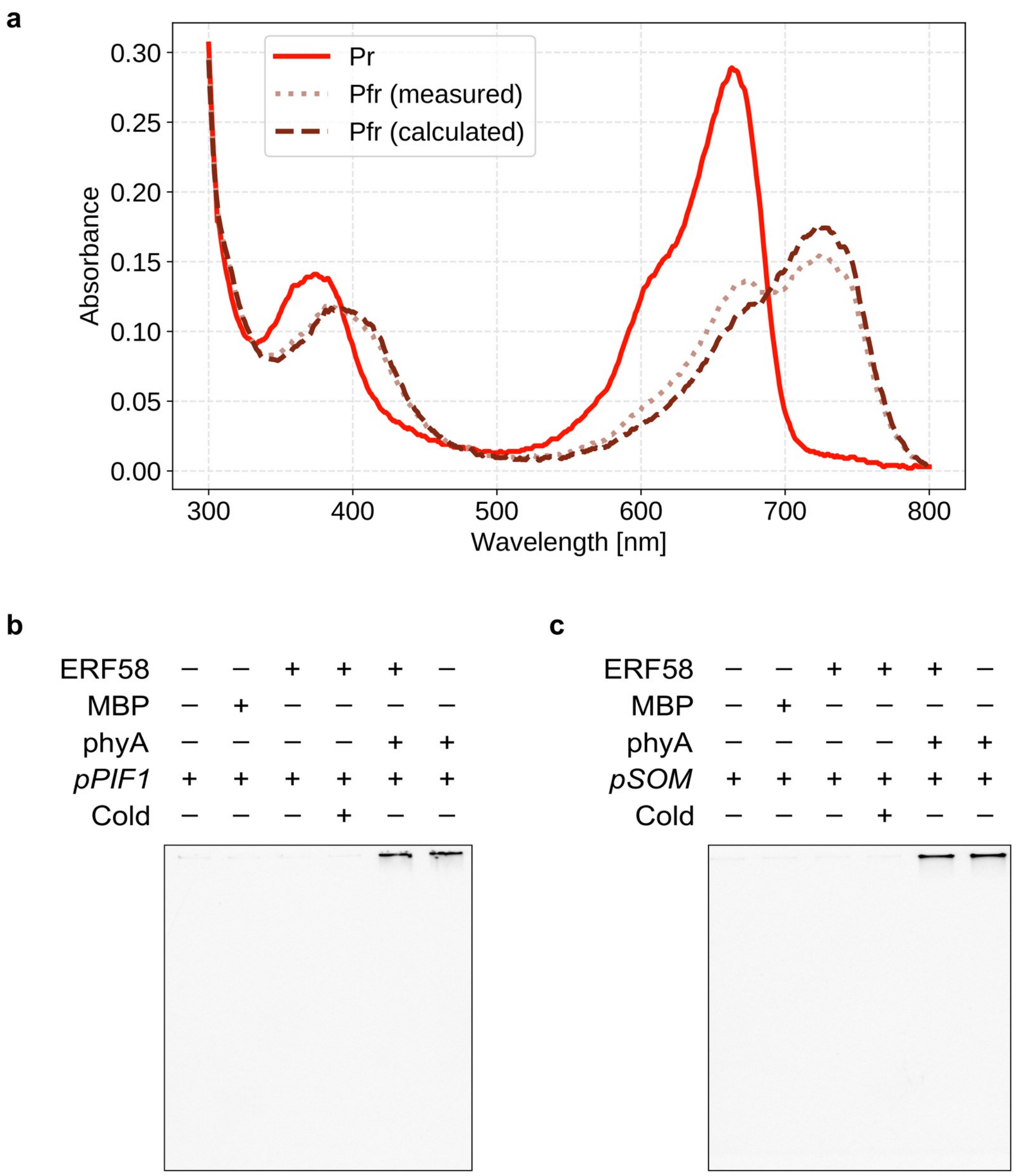

Supplementary Fig. 11 Pr/Pfr absorption spectra and zinc blot analysis for recombinant purified phyA. **a** Pr and Pfr absorption spectra of recombinant phyA. Absorption spectra of purified recombinant phyA-FLAG after saturating irradiation with far-red light (Pr) or red light (Pfr) were measured. Assuming a Pfr/Prot ratio of 0.87 after saturating irradiation with red light, the spectrum for Pfr was calculated. **b, c** Zinc blot for EMSA samples shown in **Fig. 4e, f**. Bilin-linked polypeptides in the presence of Zinc-acetate form a complex that results in orange fluorescence when viewed under UV light[1]. Here, we used this method to confirm that chromophore-bound phyA is present in the respective EMSA samples. EMSA samples were analysed on PAGE gels supplemented with Zinc-acetate and fluorescence signals were detected by exposing the PAGE gels to UV light. Cold, excess of unlabelled DNA fragment containing the DRE motif to which ERF55/ERF58 bind. The experiment was repeated 3 times with similar results.

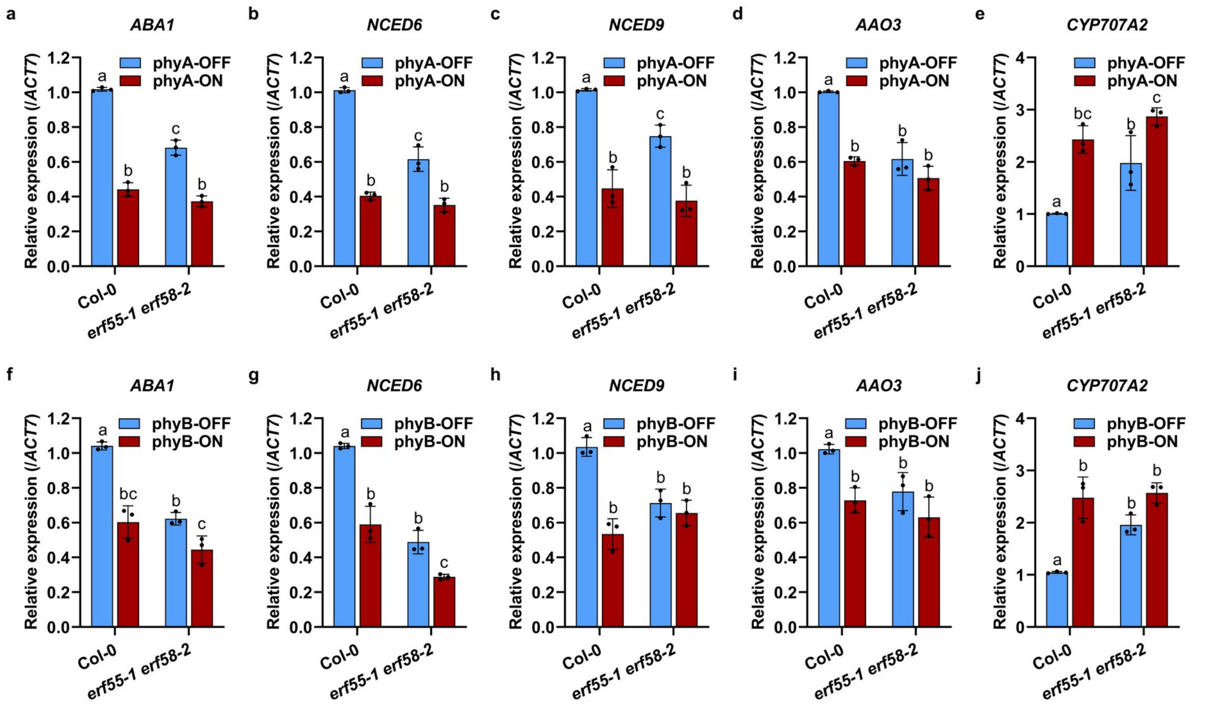

Supplementary Fig. 12 ERF55 and ERF58 regulate the expression of genes encoding ABA metabolic enzymes. **a-j** RT-qPCR was used to quantify expression of genes encoding ABA anabolic **(a-d, f-i)** or catabolic enzymes **(e, j)** in wild-type (Col-0) and *erf55-1 erf58-2* seeds germinating under phyA-ON/OFF **(a-e)** or phyB-ON/OFF conditions **(f-j)**. Light conditions are described in **Supplementary Fig. 6a, b**. *ACT7* was used as an internal control. Values show means of three replicates ±SD. Different letters indicate significant differences as determined by two-way ANOVA followed by post-hoc Tukey's HSD test; $p<0.05$.

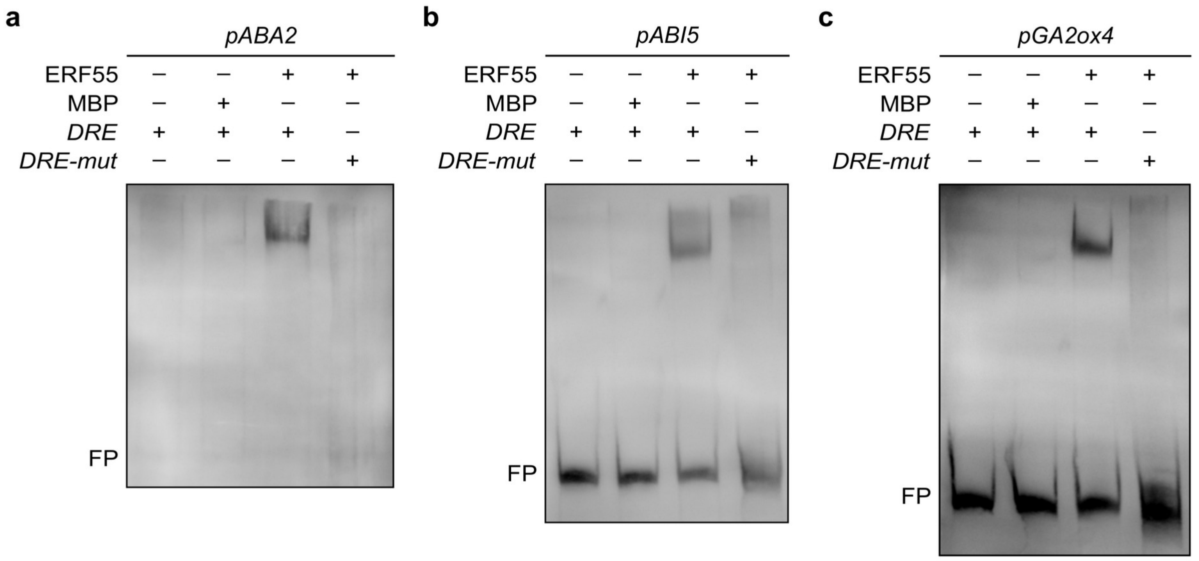

Supplementary Fig. 13 ERF55 binds to the promoter of *ABA2*, *ABI5*, and *GA2ox4*. **a-c** EMSAs. Biotin-labelled *ABA2 -52…-222* **(a)**, *ABI5 -234…-447* **(b)**, or *GA2ox4 -1111…-1264* **(c)** promoter fragments containing a wild-type or mutated DRE motif were incubated with MBP-ERF55 or MBP alone (negative control). Samples were analysed by native PAGE. Gels were blotted onto nylon membranes and signals were detected by streptavidin-coupled horseradish peroxidase and ECL. FP, free probe. Experiments were repeated six **(a)**, four **(b)**, or three times **(c)** with similar results.

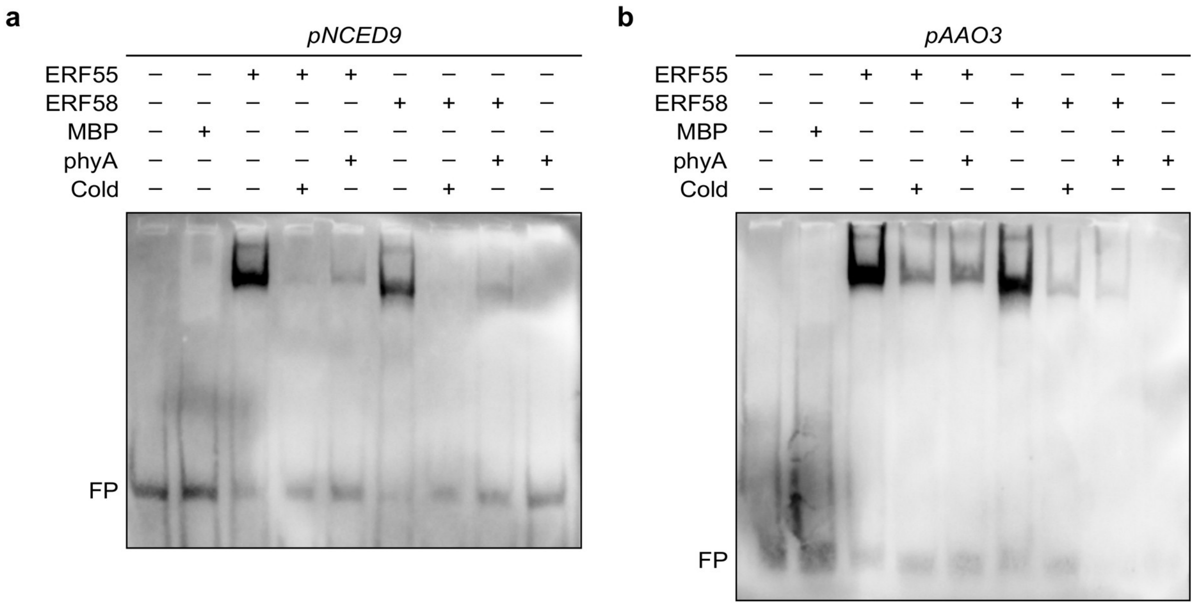

Supplementary Fig. 14 PhyA prevents the association of ERF55 and ERF58 with *NCED9* and *AAO3* promoter fragments. **a, b** EMSAs. Biotin-labelled *NCED9 -611...-633* **(a)** or *AAO3 -421...-453* **(b)** promoter fragments containing a DRE motif were incubated with MBP-ERF55, MBP-ERF58, or MBP alone (negative control). Light-activated photoactive phyA was added where indicated. Samples were analysed by native PAGE. Gels were blotted onto nylon membranes and signals were detected by streptavidin-coupled horseradish peroxidase and ECL. Cold, excess of unlabelled DNA fragment containing the DRE motif to which ERF55/ERF58 bind; FP, free probe. Experiments were repeated three times with similar results.

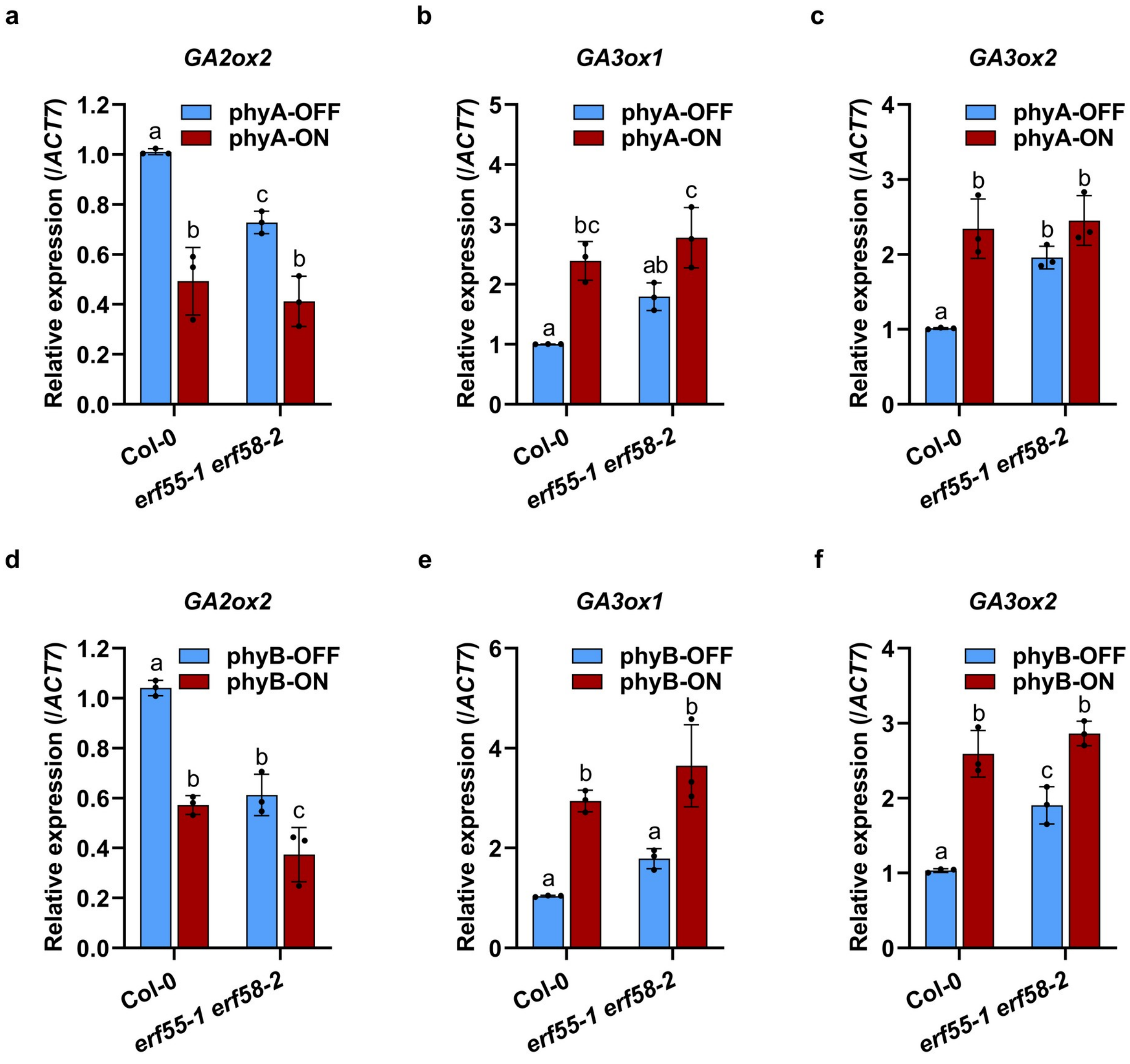

Supplementary Fig. 15 ERF55 and ERF58 regulate the expression of genes encoding GA metabolic enzymes. **a-f** RT-qPCR was used to quantify expression of genes encoding GA catabolic **(a, d)** or anabolic enzymes **(b, c, e, f)** in wild-type (Col-0) and *erf55-1 erf58-2* seeds germinating under phyA-ON/OFF **(a-c)** or phyB-ON/OFF conditions **(d-f)**. Light conditions are described in **Supplementary Fig. 6a, b**. *ACT7* was used as an internal control. Values show means of three replicates ±SD. Different letters indicate significant differences as determined by two-way ANOVA followed by post-hoc Tukey's HSD test; $p<0.05$.

a

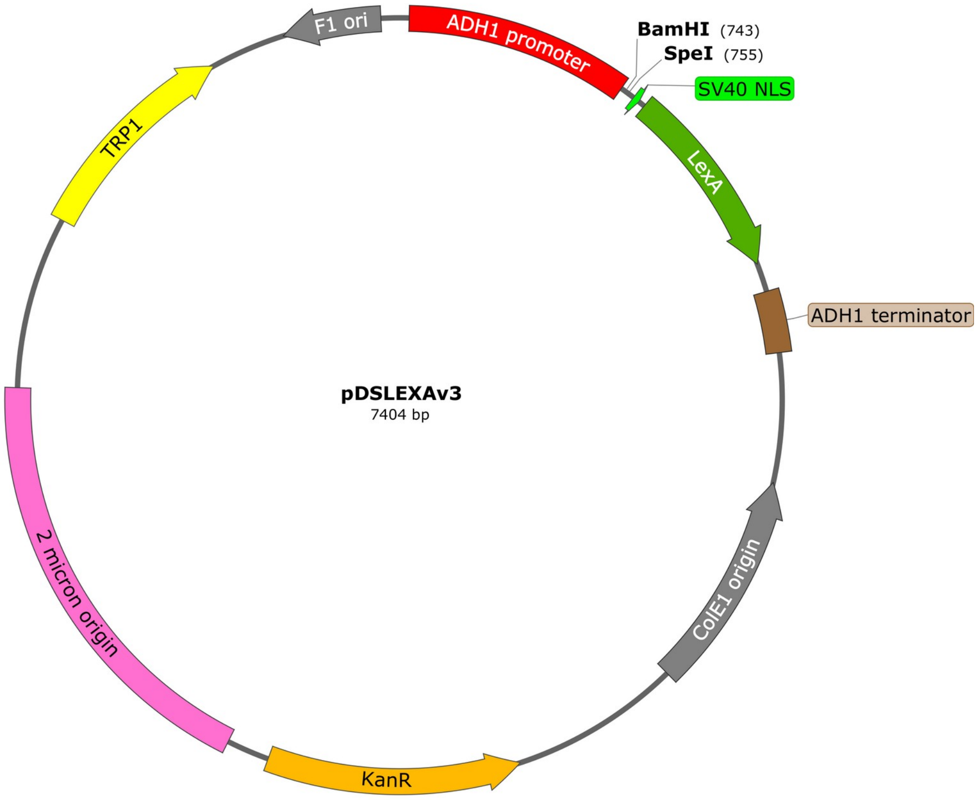

b

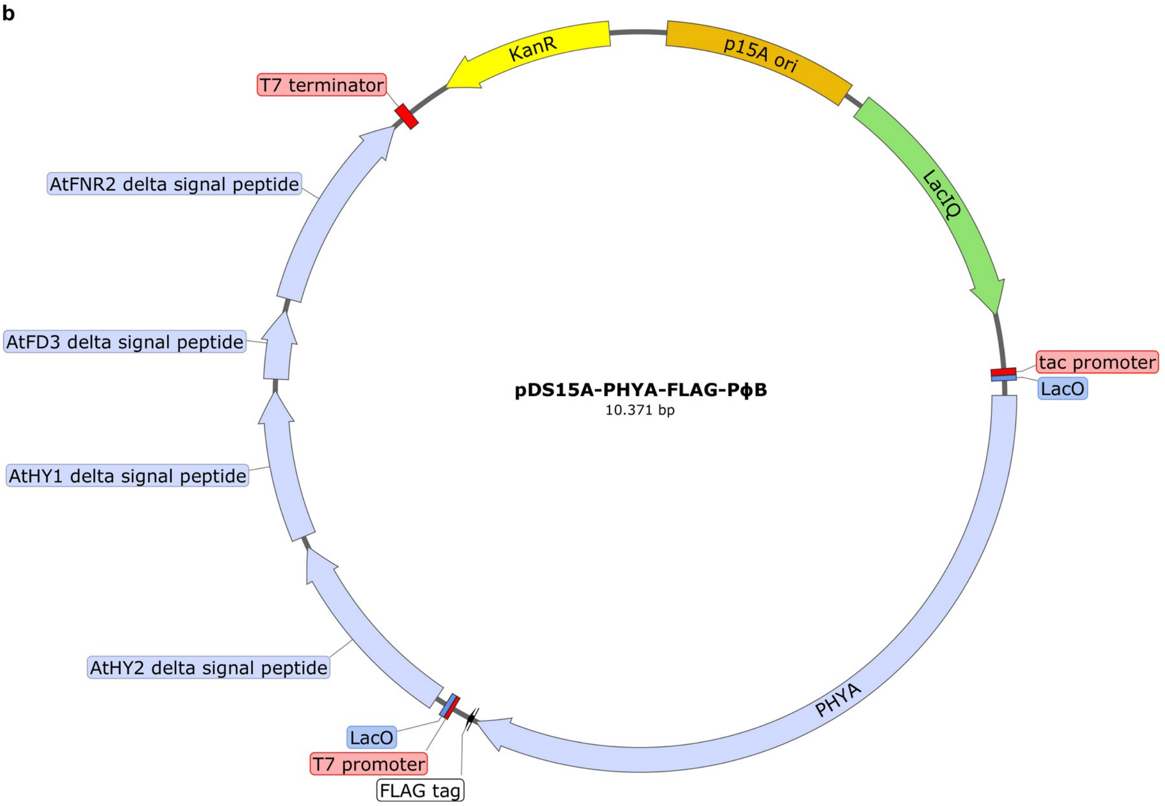

Supplementary Fig. 16 Plasmid maps for pDSLEXAv3 **(a)** and pDS15A-PHYA-FLAG-PϕB **(b)**; see **Supplementary Data 1** and **2** for nucleotide sequence.

SUPPLEMENTARY TABLES

Supplementary Table 1 Cloning of plasmid constructs.

**Name**	**Insert**	**Vector**	**Cloning strategy/reference**
pGBKMCS	*GAL4 BD*		Sheerin *et al.*[2]
pGADMCS	*GAL4 AD*		Sheerin *et al.*[2]
pDSLEXAv3	*C-term NLS-LexA (DNA-BD)*	pGBKT7 (Clontech)	See **Supplementary Data 1** and **Supplementary Fig. 16a** for nucleotide sequence and plasmid map.
pDSLEXAv3-PHYA	*PHYA-NLS-LexA*	pDSLEXAv3	The *PHYA* coding sequence was cut from pD153ah-phyA[3] using *Bam*HI/*Spe*I, and ligated into *Bam*HI/*Spe*I cut pDSLEXAv3.
pDSLEXAv3-PHYB (1-624)	*PHYB (1-624)-NLS-LexA*	pDSLEXAv3	The *PHYB PAS-GAF-PHY* coding sequence was amplified from pCHF40-PHYB using AH019/DS388, cut with *Xba*I, and ligated into *Spe*I cut pDSLEXAv3.
pGAD-ERF53	*AD-ERF53*	pGADMCS	The *ERF53* coding sequence was amplified from Arabidopsis Col-0 genomic DNA using oligos ZL005/ZL006, cut with *Bam*HI/*Xba*I, and ligated into the *Bam*HI/*Spe*I sites of pGADMCS.
pGAD-ERF54	*AD-ERF54*	pGADMCS	The *ERF54* coding sequence was amplified from Arabidopsis Col-0 genomic DNA using oligos ZL007/ZL008, cut with *Bam*HI/*Xba*I, and ligated into the *Bam*HI/*Spe*I sites of pGADMCS.
pGAD-ERF55	*AD-ERF55*	pGADMCS	The *ERF55* coding sequence was amplified from Arabidopsis Col-0 genomic DNA using oligos DS283/DS284, cut with *Bgl*II/*Xba*I, and ligated into the *Bam*HI/*Spe*I sites of pGADMCS.
pGAD-ERF56	*AD-ERF56*	pGADMCS	The *ERF56* coding sequence was amplified from Arabidopsis Col-0 genomic DNA using oligos DS285/DS286, cut with *Bam*HI/*Xba*I, and ligated into the *Bam*HI/*Spe*I sites of pGADMCS.
pGAD-ERF57	*AD-ERF57*	pGADMCS	The *ERF57* coding sequence was amplified from Arabidopsis Col-0 genomic DNA using oligos DS265/DS266, cut with *Bam*HI/*Xba*I, and ligated into the *Bam*HI/*Spe*I sites of pGADMCS.
pGAD-ERF58	*AD-ERF58*	pGADMCS	The *ERF58* coding sequence was amplified from Arabidopsis Col-0 genomic DNA using oligos DS158/DS159, cut with *Bam*HI/*Xba*I, and ligated into the *Bam*HI/*Spe*I sites of pGADMCS.
pGAD-ERF59	*AD- ERF59*	pGADMCS	The *ERF59* coding sequence was amplified from Arabidopsis Col-0 genomic DNA using oligos DS287/DS288, cut with

			*Bam*HI/*Xba*I, and ligated into the *Bam*HI/*Spe*I sites of pGADMCS.
pGAD-ERF60	*AD-ERF60*	pGADMCS	The *ERF60* coding sequence was amplified from Arabidopsis Col-0 genomic DNA using oligos DS267/DS268, cut with *Bam*HI/*Xba*I, and ligated into the *Bam*HI/*Spe*I sites of pGADMCS.
pGAD-ERF61	*AD-ERF61*	pGADMCS	The *ERF61* coding sequence was amplified from Arabidopsis Col-0 genomic DNA using oligos ZL009/ZL010, cut with *Bam*HI/*Xba*I, and ligated into the *Bam*HI/*Spe*I sites of pGADMCS.
pGAD-ERF62	*AD-ERF62*	pGADMCS	The *ERF62* coding sequence was amplified from Arabidopsis Col-0 genomic DNA using oligos ZL011/ZL012, cut with *Bam*HI/*Spe*I, and ligated into the *Bam*HI/*Spe*I sites of pGADMCS.
pGAD-ERF58 (1-77)	*AD-ERF58 (1-77)*	pGADMCS	The *ERF58 (1-77)* coding sequence was amplified from pGAD-ERF58 using oligos DS158/DS412, cut with *Bam*HI/*Xba*I, and ligated into the *Bam*HI/*Spe*I sites of pGADMCS.
pGAD-ERF58 (1-171)	*AD-ERF58 (1-171)*	pGADMCS	The *ERF58 (1-171)* coding sequence was amplified from pGAD-ERF58 using oligos DS158/DS411, cut with *Bam*HI/*Xba*I, and ligated into the *Bam*HI/*Spe*I sites of pGADMCS.
pGAD-ERF58 (78-171)	*AD-ERF58 (78-171)*	pGADMCS	The *ERF58 (78-171)* coding sequence was amplified from pGAD-ERF58 using oligos DS409/DS411, cut with *Bam*HI/*Xba*I, and ligated into the *Bam*HI/*Spe*I sites of pGADMCS.
pGAD-ERF58 (78-261)	*AD-ERF58 (78-261)*	pGADMCS	The *ERF58 (78-261)* coding sequence was amplified from pGAD-ERF58 using oligos DS409/DS159, cut with *Bam*HI/*Xba*I, and ligated into the *Bam*HI/*Spe*I sites of pGADMCS.
pGAD-ERF58 (172-261)	*AD-ERF58 (172-261)*	pGADMCS	The *ERF58 (172-261)* coding sequence was amplified from pGAD-ERF58 using oligos DS410/DS159, cut with *Bam*HI/*Xba*I, and ligated into the *Bam*HI/*Spe*I sites of pGADMCS.
pCHFv3-AvrII		pCHF5v1	pCHF5v1[4] was cut with *Nco*I/*Sal*I and ligated to annealed oligos DS358/DS359 to replace the promoter and MCS sequences; a fragment containing the terRbcS was then cut from this intermediary vector using *Sma*I/*Hind*III, blunted by fill-in, and ligated in sense orientation into the intermediary vector cut with *Sma*I.
pPPO70v1HA	*p35S:HA-EYFP*		Sheerin *et al.*[2]
pCHFv3-pERF58	*pERF58*	pCHFv3-AvrII	The -2000 promoter region of *ERF58* was

			amplified from Arabidopsis Col-0 genomic DNA using oligos DS291/DS292, cut with *Nco*I/*Xma*I, and cloned into the *Nco*I/*Xma*I sites of pCHFv3-AvrII.
pCHFv3-pERF55	*pERF55*	pCHFv3-AvrII	The -2000 promoter region of *ERF55* was amplified from Arabidopsis Col-0 genomic DNA using oligos ZL111/ZL112, cut with *Nco*I/*Bam*HI, and cloned into the *Nco*I/*Bam*HI sites of pCHFv3-AvrII.
pPPO70HA-ERF58	*p35S:HA-EYFP-ERF58*	pPPO70v1HA	The *ERF58* coding sequence was amplified from Arabidopsis Col-0 genomic DNA using oligos DS158/DS159, cut with *Bam*HI/*Xba*I, and ligated into the *Bam*HI/*Xba*I sites of pPPO70v1HA.
pPPO70HA-ERF55	*p35S:HA-EYFP-ERF55*	pPPO70v1HA	The *ERF55* coding sequence was amplified from Arabidopsis Col-0 genomic DNA using oligos DS283/DS284, cut with *Bgl*II/*Xba*I, and ligated into the *Bam*HI/*Xba*I sites of pPPO70v1HA.
pCHF-pERF58:HA-YFP-ERF58	*pERF58:HA-EYFP-ERF58*	pCHFv3-pERF58	The *ERF58* coding sequence was cut from pPPO70HA-ERF58 with *Bam*HI/*Xba*I, and ligated into the *Bam*HI/*Avr*II sites of pCHFv3-pERF58. Subsequently, HA-YFP was cut from pPPO70HA with *Xma*I/*Bam*HI, and ligated into the *Xma*I/*Bam*HI sites.
pCHF-pERF55:ERF55	*pERF55:ERF55*	pCHFv3-pERF55	PCR on genomic Arabidopsis DNA with primers DS283 and DS284 to get the *ERF55* coding sequence, cut with *Bgl*II/*Xba*I and cloned into the *Bam*HI/*Avr*II sites of pCHF-pERF55.
p35S:P19	*p35S:P19*		Chapman *et al.*[5]
pCHF40	*p35S:ECFP*		Hiltbrunner *et al.*[6]
pCHF70	*p35S:EYFP*		Hiltbrunner *et al.*[6]
pCHF40-PHYA - NLS	*p35S:PHYA-NLS-ECFP*		Sheerin *et al.*[2]
pCHF40-PHYB	*p35S:PHYB-ECFP*		Sheerin *et al.*[2]
pPPO70HA-PIF1	*p35S:HA-EYFP-PIF1*	pPPO70v1HA	The *PIF1* coding sequence was amplified from Arabidopsis Col-0 cDNA using oligos AH212/AH213, cut with *Xba*I/*Spe*I, and ligated into the *Xba*I site of pPPO70v1HA.
pPPO30HA-ABI5	*p35S:ABI5-EYFP-HA*	pPPO30HA	The *ABI5* coding sequence was amplified from ABRC cDNA clone U85657 using oligos AH764/AH765, cut with *Bam*HI/*Spe*I, and ligated into the *Bam*HI/*Xba*I sites of pPPO30v1HA[7].
pCHF5-GUS	*p35S:GUS*	pCHF5	*GUS* coding sequence was PCR amplified from pPCH1:GUS[8] using oligos DS639/DS640, cut with *Bam*HI, and cloned into the *Bam*HI site of pCHF5[6].

pCHFv3-XbaI		pCHF5v1	pCHF5v1[4] was cut with *Nco*I/*Sal*I and ligated to annealed oligos DS354/DS355 to replace the promoter and MCS sequences; a fragment containing the terRbcS was then cut from this intermediary vector using *Sma*I/*Hind*III, blunted by fill-in, and ligated in sense orientation into the intermediary vector cut with *Sma*I.
pCHF-pPIF1 (-1...-2000)-ffLUC	*pPIF1 (-1...-2000)-ffLUC*	pCHFv3-XbaI	The -1…-2000 promoter region of *PIF1* was amplified from Arabidopsis Col-0 genomic DNA using oligos ZL331/ZL320, cut with *Nco*I/*Xma*I and cloned into the *Nco*I/*Xma*I sites of pCHFv3-XbaI. The luciferase CDS was amplified from pCHF91[2] using oligos AH224/AH225, cut with *Bgl*II/*Bam*HI and cloned into the *Bam*HI site of pCHF-pPIF1(-1...-2000).
pCHF-pPIF1 (-1...-160)-ffLUC	*pPIF1 (-1...-160)-ffLUC*	pCHFv3-XbaI	The -1...-160 promoter region of *PIF1* was amplified from Arabidopsis Col-0 genomic DNA using oligos ZL342/ZL320, cut with *Nco*I/*Xma*I and cloned into the *Nco*I/*Xma*I sites of pCHFv3-XbaI. The luciferase CDS was amplified from pCHF91[2] using oligos AH224/AH225, cut with *Bgl*II/*Bam*HI and cloned into the *Bam*HI site of pCHF-pPIF1(-1...-160).
pCHF-pPIF1 (-150...-310)-ffLUC	*pPIF1 (-150...-310)-ffLUC*	pCHFv3-XbaI	The -150...-310 promoter region of *PIF1* was amplified from Arabidopsis Col-0 genomic DNA using oligos ZL340/ZL341, cut with *Nco*I/*Xma*I and cloned into the *Nco*I/*Xma*I sites of pCHFv3-XbaI. The luciferase CDS was amplified from pCHF91[2] using oligos AH224/AH225, cut with *Bgl*II/*Bam*HI and cloned into the *Bam*HI site of pCHF-pPIF1(-150...-310).
pCHF-pPIF1 (-300...-460)-ffLUC	*pPIF1 (-300...-460)-ffLUC*	pCHFv3-XbaI	The -300...-460 promoter region of *PIF1* was amplified from Arabidopsis Col-0 genomic DNA using oligos ZL338/ZL339, cut with *Nco*I/*Xma*I and cloned into the *Nco*I/*Xma*I sites of pCHFv3-XbaI. The luciferase CDS was amplified from pCHF91[2] using oligos AH224/AH225, cut with *Bgl*II/*Bam*HI and cloned into the *Bam*HI site of pCHF-pPIF1(-300...-460).
pCHF-pPIF1 (-450...-620)-ffLUC	*pPIF1 (-450...-620)-ffLUC*	pCHFv3-XbaI	The -450...-620 promoter region of *PIF1* was amplified from Arabidopsis Col-0 genomic DNA using oligos ZL336/ZL337, cut with *Nco*I/*Xma*I and cloned into the *Nco*I/*Xma*I sites of pCHFv3-XbaI. The luciferase CDS was amplified from pCHF91[2] using oligos AH224/AH225, cut with *Bgl*II/*Bam*HI and cloned into the *Bam*HI site of pCHF-pPIF1(-450...-620).
pCHF-pPIF1 (-610...-810)-ffLUC	*pPIF1 (-610...-810)-ffLUC*	pCHFv3-XbaI	The -610...-810 promoter region of *PIF1* was amplified from Arabidopsis Col-0

			genomic DNA using oligos ZL334/ZL335, cut with *Nco*I/*Xma*I and cloned into the *Nco*I/*Xma*I sites of pCHFv3-XbaI. The luciferase CDS was amplified from pCHF91[2] using oligos AH224/AH225, cut with *Bgl*II/*Bam*HI and cloned into the *Bam*HI site of pCHF-pPIF1(-610…-810).
pCHF-pPIF1 (-800...-1028)-ffLUC	*pPIF1 (-800...-1028)-ffLUC*	pCHFv3-XbaI	The -800...-1028 promoter region of *PIF1* was amplified from Arabidopsis Col-0 genomic DNA using oligos ZL321/ZL333, cut with *Nco*I/*Xma*I and cloned into the *Nco*I/*Xma*I sites of pCHFv3-XbaI. The luciferase CDS was amplified from pCHF91[2] using oligos AH224/AH225, cut with *Bgl*II/*Bam*HI and cloned into the *Bam*HI site of pCHF-pPIF1(-800…-1028).
pCHF-pPIF1 (-1…-1028)-ffLUC	*pPIF1 (-1...-1028)-ffLUC*	pCHFv3-XbaI	The -1…-1028 promoter region of *PIF1* was amplified from Arabidopsis Col-0 genomic DNA using oligos ZL321/ZL320, cut with *Nco*I/*Xma*I and cloned into the *Nco*I/*Xma*I sites of pCHFv3-XbaI. The luciferase CDS was amplified from pCHF91[2] using oligos AH224/AH225, cut with *Bgl*II/*Bam*HI and cloned into the *Bam*HI site of pCHF-pPIF1(-1…-1028).
pCHF-pSOM (-1...-2000)-ffLUC	*pSOM (-1...-2000)-ffLUC*	pCHFv3-XbaI	The -1...-2000 promoter region of *SOM* was amplified from Arabidopsis Col-0 genomic DNA using oligos ZL356/ZL328, cut with *Nco*I/*Xma*I and cloned into the *Nco*I/*Xma*I sites of pCHFv3-XbaI. The luciferase CDS was amplified from pCHF91[2] using oligos AH224/AH225, cut with *Bgl*II/*Bam*HI and cloned into the *Bam*HI site of pCHF-pSOM(-1...-2000).
pCHF-pSOM (-1...-1000)-ffLUC	*pSOM (-1...-1000)-ffLUC*	pCHFv3-XbaI	The -1...-1000 promoter region of *SOM* was amplified from Arabidopsis Col-0 genomic DNA using oligos ZL329/ZL328, cut with *Nco*I/*Xma*I and cloned into the *Nco*I/*Xma*I sites of pCHFv3-XbaI. The luciferase CDS was amplified from pCHF91[2] using oligos AH224/AH225, cut with *Bgl*II/*Bam*HI and cloned into the *Bam*HI site of pCHF-pSOM(-1...-1000).
pCHF-pSOM (-330...-510)-ffLUC	*pSOM (-330...-510)-ffLUC*	pCHFv3-XbaI	The -330...-510 promoter region of *SOM* was amplified from Arabidopsis Col-0 genomic DNA using oligos ZL363/ZL364, cut with *Nco*I/*Xma*I and cloned into the *Nco*I/*Xma*I sites of pCHFv3-XbaI. The luciferase CDS was amplified from pCHF91[2] using oligos AH224/AH225, cut with *Bgl*II/*Bam*HI and cloned into the *Bam*HI site of pCHF-pSOM(-330…-510).
pCHF-pSOM (-500...-680)-ffLUC	*pSOM (-500...-680)-ffLUC*	pCHFv3-XbaI	The -500...-680 promoter region of *SOM* was amplified from Arabidopsis Col-0 genomic DNA using oligos ZL361/ZL362,

			cut with *Nco*I/*Xma*I and cloned into the *Nco*I/*Xma*I sites of pCHFv3-XbaI. The luciferase CDS was amplified from pCHF91[2] using oligos AH224/AH225, cut with *Bgl*II/*Bam*HI and cloned into the *Bam*HI site of pCHF-pSOM(-500…-680).
pCHF-pSOM (-670...-840)-ffLUC	*pSOM (-670...-840)-ffLUC*	pCHFv3-XbaI	The -670...-840 promoter region of *SOM* was amplified from Arabidopsis Col-0 genomic DNA using oligos ZL359/ZL360, cut with *Nco*I/*Xma*I and cloned into the *Nco*I/*Xma*I sites of pCHFv3-XbaI. The luciferase CDS was amplified from pCHF91[2] using oligos AH224/AH225, cut with *Bgl*II/*Bam*HI and cloned into the *Bam*HI site of pCHF-pSOM(-670…-840).
pCHF-pSOM (-830...-1000)-ffLUC	*pSOM (-830...-1000)-ffLUC*	pCHFv3-XbaI	The -830...-1000 promoter region of *SOM* was amplified from Arabidopsis Col-0 genomic DNA using oligos ZL329/ZL358, cut with *Nco*I/*Xma*I and cloned into the *Nco*I/*Xma*I sites of pCHFv3-XbaI. The luciferase CDS was amplified from pCHF91[2] using oligos AH224/AH225, cut with *Bgl*II/*Bam*HI and cloned into the *Bam*HI site of pCHF-pSOM(-830…-1000).
pMALMCS	*MBP*		pMAL-c5X (NEB) was cut with *Nde*I/*Bam*HI and ligated to annealed oligos DS011/DS012 to replace the MCS sequence.
pMAL-ERF58	*MBP-ERF58*	pMALMCS	The *ERF58* coding sequence was amplified from Arabidopsis Col-0 genomic DNA using oligos DS158/DS159, cut with *Bam*HI/*Xba*I, and ligated into the *Bam*HI/*Spe*I sites of pMALMCS.
pMAL-ERF55	*MBP-ERF55*	pMALMCS	The *ERF55* coding sequence was amplified from Arabidopsis Col-0 genomic DNA using oligos DS283/DS284, cut with *Bgl*II/*Xba*I, and ligated into the *Bam*HI/*Spe*I sites of pMALMCS.
pMAL-FHY1 (163-202)	*MBP-FHY1 (163-202)*	pMALMCS	The *FHY1(163-202)* coding sequence was amplified from Arabidopsis Col-0 cDNA using oligos DS138/DS135, cut with *Bam*HI/*Spe*I, and ligated into the *Bam*HI/*Spe*I sites of pMALMCS.
pDS15A-PHYA-FLAG-PϕB	*PHYA-FLAG, AtHY2, AtHY1, AtFD3, AtFNR2*		See **Supplementary Data 2** and **Supplementary Fig. 16b** for nucleotide sequence and plasmid map.

Supplementary Table 2 Primers used for cloning of plasmid constructs.

AH019	CCCAAGCTTCTAGAAAAATGGTTTCCGGAGTCGGG
AH212	GCTCTAGAAAAATGCATCATTTTGTCCCTG
AH213	GGACTAGTACCTGTTGTGTGGTTTCCG
AH224	GAAGATCTAAAAATGGAAGACGCCAAAAACA
AH225	GGACTAGTTATCTAGAGCTTACCTAGGATCCGCCACGGCGATCTTTCCGCCC
AH764	CGCGGATCCAAAAATGGTAACTAGAGAAACG
AH765	GGACTAGTGAGTGGACAACTCGGGTT
DS011	TATGGATCCCGGGACTAGTTAAA
DS012	GATCTTTAACTAGTCCCGGGATCCA
DS135	ACGTCACTAGTTTACAGCATTAGCGTTGAGAAGT
DS138	ACGTCGGATCCAGGAATGGCTGCTAAGTTTGTTCTGTCCTC
DS158	ACGTCGGATCCAGGAATGACAACTTCTATGGATTTTTACA
DS159	ACGTCTCTAGAGCCATTTACAAGACTCGAACACTGAA
DS265	ACGTCGGATCCAGGAATGGCTTTAAACATGAATGCTTAC
DS266	ACGTCTCTAGAGAAGAGTTTCTCTATAGCGTC
DS267	ACGTCGGATCCAGGAATGGCAGCCATAGATATGTTC
DS268	ACGTCTCTAGAAGATTCGGACAATTTGCTAATC
DS283	ACGTCAGATCTAGGAATGGCGGATCTCTTCGGTGGT
DS284	ACGTCTCTAGAGCCCGATAAAATTGAAGCCCAATCT
DS285	ACGTCGGATCCAGGAATGGAAACTGCTTCTCTTTCTTTC
DS286	ACGTCTCTAGAGCCAGAATTGGCCAGTTTACTAATTG
DS287	ACGTCGGATCCAGGAATGGCAGCTGCTATGAATTTGT
DS288	ACGTCTCTAGAGCCAGCTAGAATCGAATCCCAATC
DS291	ACGTCCCATGGTATTTCATTTCCCCTGTGGACACGC
DS292	ACGTCCCCGGGTTCTAATAAAAAGAAATTTTTTTCC
DS354	CATGGTACCCGGGGCGGATCCAAAATCTAGATAAG
DS355	TCGACTTATCTAGATTTTGGATCCGCCCCGGGTAC
DS358	CATGGTACCCGGGGCGGATCCAAAACCTAGGTAAG
DS359	TCGACTTACCTAGGTTTTGGATCCGCCCCGGGTAC
DS388	ACGTCTCTAGACGCAGCCGCCTCAGATTCTTTAA
DS409	ACGTCGGATCCAGGAATGAAACCGACGAAGCTATACAGAG
DS410	ACGTCGGATCCAGGAATGCAGGTGAGATCAACGAAGAAGT
DS411	ACGTCTCTAGAGCCTTTCTGCGTCGTCTCAGCTAA
DS412	ACGTCTCTAGAGCCAGCCGCGGAAGTACCGGTT
DS639	ACGTCGGATCCAACAATGTTACGTCCTGTAGAAACCCCAA
DS640	ACGTCGGATCCTTATTGTTTGCCTCCCTGCTGC
ZL005	ACGTCGGATCCAGGAATGGCTACTGCTAAGAACAAGG

ZL006	ACGTCTCTAGACTAAATTGTATCAGAAGAAGAGTT
ZL007	ACGTCGGATCCAGGAATGGACTTTGACGAGGAG
ZL008	ACGTCTCTAGATTCAAAAGAAAGGCCTCATAGGA
ZL009	ACGTCGGATCCAGGAATGGAAGAAAGCAATGATATTTTTC
ZL010	ACGTCTCTAGATTAATTGGCAAGAACTTCCCAAA
ZL011	ACGTCGGATCCAGGAATGATCACACCAATACACACAC
ZL012	ACGTCACTAGTTCAAGAAGAATGAGGAAATGAGA
ZL111	ACGTCCCATGGTCTCTAAAACATCTTAAAGTGAAAC
ZL112	ACGTCGGATCCGGAGTTTCCTGAAATACAAAAAA
ZL320	ACGTCCCCGGGATCTCTCTCTCTACAAAGATGAT
ZL321	ACGTCCCATGGACTCCTCTAGAATTCGAGGCT
ZL328	ACGTCCCCGGGGATGATATAATATCTAACTTGTTTTTTTTG
ZL329	ACGTCCCATGGAATTTCGTTATATTTGTTATTTCGTC
ZL331	ACGTCCCATGGAATTATATAAAAATATGTATATACATATTAATG
ZL333	ACGTCCCCGGGTTAAACATAACTGAATCGCATGTTA
ZL334	ACGTCCCATGGGTTATGTTTAAGTGTTAATCAGTTA
ZL335	ACGTCCCCGGGAGCCTCATTGTTTCTCATCCGT
ZL336	ACGTCCCATGGACAATGAGGCTGATTATTTTATCT
ZL337	ACGTCCCCGGGTGTGTCAACTAACTTTAACAGTGA
ZL338	ACGTCCCATGGTAGTTGACACAATAGTAAATGCG
ZL339	ACGTCCCCGGGTGTGCTAAAGGATGACTGAATC
ZL340	ACGTCCCATGGCCTTTAGCACAAAAAAAAAAAAAAAAAG
ZL341	ACGTCCCCGGGTAATCAGTGAAGAAACATATATACT
ZL342	ACGTCCCATGGTTCACTGATTAGTAATCTCTGTG
ZL356	ACGTCCCATGGTTGTATATATAAGTAATCATATCACTA
ZL358	ACGTCCCCGGGAAAATAACGTAGTACATAACCGAAT
ZL359	ACGTCCCATGGTACGTTATTTTACATAGCAGTTACGTATCCA
ZL360	ACGTCCCCGGGAGTTTTACTTATTTTTTCATATATTAATTTG
ZL361	ACGTCCCATGGTAAGTAAAACTGCTTTAAATAATAATC
ZL362	ACGTCCCCGGGTTGTCTTCTAGTTTGTTACCAAG
ZL363	ACGTCCCATGGCTAGAAGACAAATTCATACATTA
ZL364	ACGTCCCCGGGTTGCTTCAGACCTAACACATGTGC

Supplementary Table 3 Genotyping of mutants used in this study.

**Allele**	**NASC ID/reference**	**Genotyping protocol**
phyA-211	NASC N6223; Reed *et al.*[9]	PCR with primers PHYA fw and PHYA rev amplifies WT allele, QRT1 fw and PHYA rev amplifies *phyA-211*.
phyB-9	NASC N6217; Reed *et al.*[10]	PCR with primers PHYB fw and PHYB rev; then sequence the product; the *phyB-9* mutant contains a premature stop codon.
pif1-1	NASC N66041; SAIL_256_G07	PCR with primers PIF1 fw and PIF1 rev amplifies WT allele, PIF1 rev and SAIL LB3 amplifies *pif1-1*.
erf58-1	NASC N677156; SALK_139727	PCR with primers ERF58 fw and ERF58 rev amplifies WT allele, ERF58 rev and SALK LBb1.3 amplifies *erf58-1*.
erf58-2	NASC N848391; SAIL_1293_A03	PCR with primers ERF58 fw and ERF58 rev amplifies WT allele, ERF58 rev and SAIL LB3 amplifies *erf58-2*.
erf55-1	NASC N681465; SALK_091212	PCR with primers ERF55 fw and ERF55 rev amplifies WT allele, ERF55 fw and SALK LBb1.3 amplifies *erf55-1*.
erf56	NASC N661174; SALK_139786	PCR with primers ERF56 fw and ERF56 rev amplifies WT allele, ERF56 rev and SALK LBb1.3 amplifies *erf56*.
erf57	NASC N409378; GK-098F06	PCR with primers ERF57 fw and ERF57 rev amplifies WT allele, ERF57 rev and GABI KAT RB o3144 amplifies *erf57*.
erf59	NASC N654090; SALK_020767	PCR with primers ERF59 fw and ERF59 rev amplifies WT allele, ERF59 rev and SALK LBb1.3 amplifies *erf59*.
erf60	NASC N656258; SALK_021999	PCR with primers ERF60 fw and ERF60 rev amplifies WT allele, ERF60 rev and SALK LBb1.3 amplifies *erf60*.
abi5-8	NASC N673861; SALK_013163C	PCR with primers ABI5 fw and ABI5 rev amplifies WT allele, ABI5 rev and SALK LBb1.3 amplifies *abi5-8*.
som-3	NASC N508075; SALK_008075	PCR with primers SOM fw and SOM rev amplifies WT allele, SOM rev and SALK LBb1.3 amplifies *som-3*.

Supplementary Table 4 Primers for genotyping of mutants used in this study.

SALK LBb1.3	DS274	ATTTTGCCGATTTCGGAAC
SAIL LB3	DS275	TAGCATCTGAATTTCATAACCAATCTCGATACAC
GABI KAT RB o3144	DS277	GTGGATTGATGTGATATCTCC
ERF58 fw	DS241	ACGTCGGATCCAGGAATGACAACTTCTATGGATTTTTAC
ERF58 rev	DS242	ACGTCTCTAGAATTTACAAGACTCGAACACTGAA
ERF55 fw	DS243	ATGGCGGATCTCTTCGGTGG
ERF55 rev	DS244	CGATAAAATTGAAGCCCAATCT
ERF56 fw	DS247	AAAGTAAAACATGTCTAAATTCAC
ERF56 rev	DS248	AGAATTGGCCAGTTTACTAATTG
ERF57 fw	DS265	ACGTCGGATCCAGGAATGGCTTTAAACATGAATGCTTAC
ERF57 rev	DS266	ACGTCTCTAGAGAAGAGTTTCTCTATAGCGTC
ERF59 fw	DS287	ACGTCGGATCCAGGAATGGCAGCTGCTATGAATTTGT
ERF59 rev	DS288	ACGTCTCTAGAGCCAGCTAGAATCGAATCCCAATC
ERF60 fw	DS327	CATGGAATAAAAGATTTTTATCAAG
ERF60 rev	DS268	ACGTCTCTAGAAGATTCGGACAATTTGCTAATC
PIF1 fw	AH678	CATGAATTTCTCGAGGCTGAG
PIF1 rev	AH677	AAGGAAGGAGGAGGAATAGGC
SOM fw	ZL363	ACGTCCCATGGCTAGAAGACAAATTCATACATTA
SOM rev	ZL414	ACAATCAGTCCAATCGTGGCTT
ABI5 fw	ZL354	ACGTCCCATGGCTTTCACCAGCTAGAAGCTCAA
ABI5 rev	ZL347	ACGTCCCCGGGCCGCCGGAAAACTGAGAGAA
PHYA fw	AH861	ACACGATGATTCCTGCATCTG
PHYA rev	AH862	AGCTGTGCGGTGCTCTAAG
QRT1 fw	DS547	TTGAAGCTAAATTCTTGGGTTAGT
PHYB fw	DS799	GTGGAAAGTGTGAGGGACTTGA
PHYB rev	DS942	CACTAAGTCCATGATACTGGGAC

Supplementary Table 5 Primers used for qPCR.

ACTIN1 fw	BE76	CATCAGGAAGGACTTGTACGG	Enderle *et al.*[8]
ACTIN1 rev	BE77	CATGGACTTGACTCGTCATAC	Enderle *et al.*[8]
ACTIN7 fw	ZL273	GTTGCCATTCAGGCCGTTCTTTC	Rudnik *et al.*[11]
ACTIN7 rev	ZL274	GAGAATCGAGCACAATACCGGTTG	Rudnik *et al.*[11]
GA2ox2 fw	ZL181	TCCTAAAACCTCCGCCGTTT	Jasinski *et al.*[12]
GA2ox2 rev	ZL182	CCGACTCACGGAAAATTTGAG	Jasinski *et al.*[12]
GA2ox4 fw	ZL183	TCAGCTCGGCAGTGAATTGT	Jasinski *et al.*[12]
GA2ox4 rev	ZL184	GAAGTCCCTCAGCCGTCAGAT	Jasinski *et al.*[12]
PIF1 fw	ZL187	GATCCTCAGCAGCAACCTTCTTC	Zhong *et al.*[13]
PIF1 rev	ZL188	ACCTGCGTAGATTCTCTCACAACC	Zhong *et al.*[13]
ABI5 fw	ZL281	CAGCTGCAGGTTCACATTCT	this study
ABI5 rev	ZL282	TCGTTCGCTATCCCTCTCTT	this study
NCED6 fw	ZL283	TTGAGTCCTCTATCGTTATTCCTATGG	Shang *et al.*[14]
NCED6 rev	ZL284	GGAGCGAAGTTACCTGATAATTGAA	Shang *et al.*[14]
NCED9 fw	ZL285	GGAAAACGCCATGATCTCACA	Lee *et al.*[15]
NCED9 rev	ZL286	GCAGGATCCGCCGTTTTAG	Lee *et al.*[15]
SOM fw	ZL289	TCCGGATGTTCGAATTCAAGAT	Lee *et al.*[15]
SOM rev	ZL290	GCAAAAGGACAATCAGTCCAATC	Lee *et al.*[15]
ABA2 fw	ZL293	ACTCGCTTTGGCTCATTTGCC	Yu *et al.*[16]
ABA2 rev	ZL294	ACCGTCAGTTCCACCCCTTT	Yu *et al.*[16]
ABA1 fw	ZL297	AGCAGATTCTAGCACGTGCGGTT	this study
ABA1 rev	ZL298	CATCTGCACCCACAAGCAGAT	this study
GA3ox1 fw	ZL301	CCATTCACCTCCCACACTCT	Lee *et al.*[17]
GA3ox1 rev	ZL302	GCCAGTGATGGTGAAACCTT	Lee *et al.*[17]
GA3ox2 fw	ZL303	CTGCCGCTCATCGACCTC	Lee *et al.*[17]
GA3ox2 rev	ZL304	AGCATGGCCCACAAGAGTG	Lee *et al.*[17]
CYP707A2 fw	ZL299	AGAGAAGCAGTACAAGACGTCG	Lee *et al.*[17]
CYP707A2 rev	ZL300	ACATGAGTGCACTCCATTTCCG	Lee *et al.*[17]
ERF58 fw	ZL441	ATCTCCACTCATTTTCTCACCAT	this study
ERF58 rev	ZL442	GTGTCGAATGTTCCGAGCCAAAGT	this study
ERF55 fw	ZL443	ACATTCCACAACCTCTTACCC	this study

ERF55 rev	ZL444	TCGGCTCAATGAAGTTAAGCAT	this study
ERF58-rbcS fw	ZL504	ATTCGATTCTTCAGTGTTCGAGT	this study
ERF58-rbcS rev	DS76	TCGAACTCAGTAGGATTCTG	this study
ERF55-rbcS fw	ZL503	AAGTGTCCTTCTTATGAGATAGA	this study
ERF55-rbcS rev	DS76	TCGAACTCAGTAGGATTCTG	this study
AAO3 fw	ZL529	GAAGGTCTTGGAAACACGAAGAA	Jiang *et al.*[18]
AAO3 rev	ZL530	GAAATACACATCCCTGGTGTACAAAAC	Jiang *et al.*[18]

Supplementary Table 6 Probes for EMSA experiments.

Promoter	Region	Strategy
pSOM	-621...-840	PCR on Arabidopsis Col-0 gDNA with primers ZL411 and ZL384; the product was purified by gel extraction kit.
pPIF1	-801...-1031	PCR on Arabidopsis Col-0 gDNA with primers ZL405 and ZL388; the product was purified by gel extraction kit.
pABA2-DRE	-52...-222	PCR on Arabidopsis Col-0 gDNA with primers ZL480 and ZL471; the product was purified by gel extraction kit.
pABA2-DRE-mut	-52...-222	PCR on Arabidopsis Col-0 gDNA with primers ZL480 and ZL470 to get fragment 1 and PCR on gDNA with primers ZL469 and ZL471 to get fragment 2; then fuse the two fragments by overlap extension PCR using primers ZL480 and ZL471; the product was purified by gel extraction kit.
pAAO3	-421...-453	Anneal complementary oligonucleotides ZL523 and ZL524.
pNCDE9	-611...-633	Anneal complementary oligonucleotides ZL521 and ZL522.
pABI5-DRE	-234...-447	PCR on gDNA with primers ZL475 and ZL476; the product was purified by gel extraction kit.
pABI5-DRE-mut	-234...-447	PCR on Arabidopsis Col-0 gDNA with primers ZL475 and ZL478 to get fragment 1 and PCR on gDNA with primers ZL477 and ZL476 to get fragment 2; then fuse the two fragments by overlap extension PCR using primers ZL475 and ZL476; the product was purified by gel extraction kit.
pGA2ox4-DRE	-1111...-1346	PCR on Arabidopsis Col-0 gDNA with primers ZL472 and ZL434; the product was purified by gel extraction kit.
pGA2ox4-DRE-mut	-1111...-1346	PCR on Arabidopsis Col-0 gDNA with primers ZL472 and ZL474 to get fragment 1 and PCR on gDNA with primers ZL473 and ZL434 to get fragment 2; then fuse the two fragments by overlap extension PCR using primers ZL472 and ZL434; the product was purified by gel extraction kit.

Supplementary Table 7 Primers used for generation of probes for EMSA experiments.

DRE box fw	DS980	TTGATACTACCGACATGAGTTGATACTACCGACATGAGTT
DRE box rev	DS979	AACTCATGTCGGTAGTATCAACTCATGTCGGTAGTATCAA
SOM promoter fw	ZL411	TACGTTATTTTACATAGCAGTTAC–Biotin
SOM promoter rev	ZL384	TCAGCATTTGAAGTATTATTTTTCC
PIF1 promoter fw	ZL405	AATACTCCTCTAGAATTCGAGGCT–Biotin
PIF1 promoter rev	ZL388	TAAACATAACTGAATCGCATGTTACCAGATTACCAT
ABA2 promoter fw	ZL480	ATCCGTTAGTCTTTTATTGGGCT–Biotin
ABA2 promoter rev	ZL471	ACTATTGACACAGACAAGCTCA
ABA2 promoter-mut fw	ZL469	TAGAGACTTATACCAAATAGCCGAGAAACAACGCA
ABA2 promoter-mut rev	ZL470	TGCGTTGTTTCTCGGCTATTTGGTATAAGTCTCTA
GA2ox4 promoter fw	ZL472	AGTCTGGCTACAAAGCAGAAC–Biotin
GA2ox4 promoter rev	ZL434	TCTTGAAACTGCAAAGAGATGG
GA2ox4 promoter-mut fw	ZL473	AAACTTGACTTTATCAAATTCAAACCCATGTTATTTTTATCTA
GA2ox4 promoter-mut rev	ZL474	TAGATAAAAATAACATGGGTTTGAATTTGATAAAGTCAAGTTT
ABI5 promoter fw	ZL475	AACGAAAACTTTATTGGAAGAGTA–Biotin
ABI5 promoter rev	ZL476	TGATAATTGATGCTCTCAAAGAATG
ABI5 promoter-mut fw	ZL477	TCACGTGGATTTATTTAATTACCTTCTTTCATTCTTGTTA
ABI5 promoter-mut rev	ZL478	TAACAAGAATGAAAGAAGGTAATTAAATAAATCCACGTGA
AAO3 promoter fw	ZL523	AGTAGTTTTTTTTTTGTCGGCATCACTAGTAGT–Biotin
AAO3 promoter rev	ZL524	ACTACTAGTGATGCCGACAAAAAAAAAAACTACT
NCED9 promoter fw	ZL521	ACTCAATAATACCGACAGTCTCA–Biotin
NCED9 promoter rev	ZL522	TGAGACTGTCGGTATTATTGAGT

Supplementary Table 8 Primers used for ChIP-qPCR.

ABI5 promoter fw	ZL481	AACGAAAACTTTATTGGAAGAGTA
ABI5 promoter rev	ZL476	TGATAATTGATGCTCTCAAAGAATG
SOM promoter fw	ZL383	TACGTTATTTTACATAGCAGTTAC
SOM promoter rev	ZL384	TCAGCATTTGAAGTATTATTTTTCC
PIF1 promoter fw	ZL387	AATACTCCTCTAGAATTCGAGGCT
PIF1 promoter rev	ZL388	TAAACATAACTGAATCGCATGTTACCAGATTACCAT
ABA2 promoter fw	ZL429	ATCGGTCTAACTTAACGTCTCTA
ABA2 promoter rev	ZL471	ACTATTGACACAGACAAGCTCA
GA2ox4 promoter fw	ZL433	ACCATGTTCCATATATATGAATGTAC
GA2ox4 promoter rev	ZL434	TCTTGAAACTGCAAAGAGATGG

SUPPLEMENTARY REFERENCES

1. Berkelman, T. R. & Lagarias, J. C. Visualization of bilin-linked peptides and proteins in polyacrylamide gels. *Anal. Biochem.* **156**, 194–201 (1986).
2. Sheerin, D. J. *et al.* Light-activated phytochrome A and B interact with members of the SPA family to promote photomorphogenesis in Arabidopsis by reorganizing the COP1/SPA complex. *Plant Cell* **27**, 189–201 (2015).
3. Rausenberger, J. *et al.* Photoconversion and nuclear trafficking cycles determine phytochrome A's response profile to far-red light. *Cell* **146**, 813–825 (2011).
4. Menon, C., Klose, C. & Hiltbrunner, A. Arabidopsis FHY1 and FHY1-LIKE are not required for phytochrome A signal transduction in the nucleus. *Plant Comm.* **1**, 100007 (2020).
5. Chapman, E. J., Prokhnevsky, A. I., Gopinath, K., Dolja, V. V. & Carrington, J. C. Viral RNA silencing suppressors inhibit the microRNA pathway at an intermediate step. *Genes Dev.* **18**, 1179–1186 (2004).
6. Hiltbrunner, A. *et al.* Nuclear accumulation of the phytochrome A photoreceptor requires FHY1. *Curr. Biol.* **15**, 2125–2130 (2005).
7. Possart, A. *et al.* Characterization of phytochrome interacting factors from the moss *Physcomitrella patens* illustrates conservation of phytochrome signaling modules in land plants. *Plant Cell* **29**, 310–330 (2017).
8. Enderle, B. *et al.* PCH1 and PCHL promote photomorphogenesis in plants by controlling phytochrome B dark reversion. *Nat. Commun.* **8**, 2221 (2017).
9. Reed, J. W., Nagatani, A., Elich, T. D., Fagan, M. & Chory, J. Phytochrome A and phytochrome B have overlapping but distinct functions in *Arabidopsis* development. *Plant Physiol.* **104**, 1139–1149 (1994).
10. Reed, J. W., Nagpal, P., Poole, D. S., Furuya, M. & Chory, J. Mutations in the gene for the red/far-red light receptor phytochrome B alter cell elongation and physiological responses throughout *Arabidopsis* development. *Plant Cell* **5**, 147–157 (1993).
11. Rudnik, R., Bulcha, J. T., Reifschneider, E., Ellersiek, U. & Baier, M. Specificity versus redundancy in the RAP2.4 transcription factor family of *Arabidopsis thaliana*: transcriptional regulation of genes for chloroplast peroxidases. *BMC Plant Biol.* **17**, 144 (2017).
12. Jasinski, S. *et al.* KNOX action in *Arabidopsis* is mediated by coordinate regulation of cytokinin and gibberellin activities. *Curr. Biol.* **15**, 1560–1565 (2005).
13. Zhong, S. *et al.* A molecular framework of light-controlled phytohormone action in Arabidopsis. *Curr. Biol.* **22**, 1530–1535 (2012).
14. Shang, Y., Fu, M. & Nam, K. H. Suppressor of *bri1-120* mutant allele revealed interrelated and independent actions of brassinosteroid and light signaling. *J. Plant Biol.* **59**, 594–602 (2016).
15. Lee, K. P. *et al.* Spatially and genetically distinct control of seed germination by phytochromes A and B. *Genes Dev.* **26**, 1984–1996 (2012).

16. Yu, L.-H. *et al.* Arabidopsis MADS-Box transcription factor AGL21 acts as environmental surveillance of seed germination by regulating *ABI5* expression. *Mol. Plant* **10**, 834–845 (2017).
17. Lee, S. A. *et al.* Interplay between ABA and GA modulates the timing of asymmetric cell divisions in the Arabidopsis root ground tissue. *Mol. Plant* **9**, 870–884 (2016).
18. Jiang, Z., Xu, G., Jing, Y., Tang, W. & Lin, R. Phytochrome B and REVEILLE1/2-mediated signalling controls seed dormancy and germination in Arabidopsis. *Nat. Commun.* **7**, 12377 (2016).
