# Supplementary material for: The phytochrome interacting proteins ERF55 and ERF58 repress light-induced seed germination in Arabidopsis thaliana
Source: Nat Commun. 2022 Mar 29;13:1656. doi: 10.1038/s41467-022-29315-3 (PMC8964797; doi:10.1038/s41467-022-29315-3)
Supplement: Supplementary file 3 — Description of Additional Supplementary Files [file 41467_2022_29315_MOESM3_ESM.pdf]

Description of Additional Supplementary Files

File name: Supplementary Data 1

Description: **Title: pDSLEXAv3**

◦ Legend: Genbank file containing nucleotide sequence and annotation for vector pDSLEXAv3.

File name: Supplementary Data 2

Description: **Title: pDS15A-PHYA-FLAG-PϕB**

◦ Legend: Genbank file containing nucleotide sequence and annotation for vector pDS15A-PHYA-FLAG-PϕB.
