# Supplementary material for: The phytochrome interacting proteins ERF55 and ERF58 repress light-induced seed germination in Arabidopsis thaliana
Source: Nat Commun. 2022 Mar 29;13:1656. doi: 10.1038/s41467-022-29315-3 (PMC8964797; doi:10.1038/s41467-022-29315-3)
Supplement: Supplementary file 6 — Reporting Summary [file 41467_2022_29315_MOESM6_ESM.pdf]

nature portfolio

Corresponding author(s): Andreas Hiltbrunner

Last updated by author(s): 2021-12-22

# Reporting Summary

Nature Portfolio wishes to improve the reproducibility of the work that we publish. This form provides structure for consistency and transparency in reporting. For further information on Nature Portfolio policies, see our Editorial Policies and the Editorial Policy Checklist.

## Statistics

For all statistical analyses, confirm that the following items are present in the figure legend, table legend, main text, or Methods section.

| n/a | Confirmed | |
|---|---|---|
| ☐ | ☒ | The exact sample size (*n*) for each experimental group/condition, given as a discrete number and unit of measurement |
| ☐ | ☒ | A statement on whether measurements were taken from distinct samples or whether the same sample was measured repeatedly |
| ☐ | ☒ | The statistical test(s) used AND whether they are one- or two-sided<br>*Only common tests should be described solely by name; describe more complex techniques in the Methods section.* |
| ☒ | ☐ | A description of all covariates tested |
| ☒ | ☐ | A description of any assumptions or corrections, such as tests of normality and adjustment for multiple comparisons |
| ☐ | ☒ | A full description of the statistical parameters including central tendency (e.g. means) or other basic estimates (e.g. regression coefficient) AND variation (e.g. standard deviation) or associated estimates of uncertainty (e.g. confidence intervals) |
| ☐ | ☒ | For null hypothesis testing, the test statistic (e.g. *F*, *t*, *r*) with confidence intervals, effect sizes, degrees of freedom and *P* value noted<br>*Give P values as exact values whenever suitable.* |
| ☒ | ☐ | For Bayesian analysis, information on the choice of priors and Markov chain Monte Carlo settings |
| ☒ | ☐ | For hierarchical and complex designs, identification of the appropriate level for tests and full reporting of outcomes |
| ☒ | ☐ | Estimates of effect sizes (e.g. Cohen's *d*, Pearson's *r*), indicating how they were calculated |

*Our web collection on statistics for biologists contains articles on many of the points above.*

## Software and code

Policy information about availability of computer code

| | |
|---|---|
| Data collection | - Epifluorescence microscope: MetaMorph Version 5.0r2<br>- Luminescence/Fluorescence plate reader: Omega BMG Labtech<br>- RT-qPCR system: CFX384 Touch real-Time PCR Detection System (Bio-Rad Laboratories) |
| Data analysis | - ImageJ version 1.53n<br>- MARS Data Analysis Software Program version 3.10 R6, BMG Labtech<br>- GraphPad Prism Version 9.2.0 |

For manuscripts utilizing custom algorithms or software that are central to the research but not yet described in published literature, software must be made available to editors and reviewers. We strongly encourage code deposition in a community repository (e.g. GitHub). See the Nature Portfolio guidelines for submitting code & software for further information.

## Data

Policy information about availability of data

All manuscripts must include a data availability statement. This statement should provide the following information, where applicable:
- Accession codes, unique identifiers, or web links for publicly available datasets
- A description of any restrictions on data availability
- For clinical datasets or third party data, please ensure that the statement adheres to our policy

All data generated or analysed during this study are included in this published article (and its supplementary information files).

# Field-specific reporting

Please select the one below that is the best fit for your research. If you are not sure, read the appropriate sections before making your selection.

☒ Life sciences ☐ Behavioural & social sciences ☐ Ecological, evolutionary & environmental sciences

For a reference copy of the document with all sections, see nature.com/documents/nr-reporting-summary-flat.pdf

# Life sciences study design

All studies must disclose on these points even when the disclosure is negative.

| | |
|---|---|
| Sample size | - Y2H liquid assay: 3 biological replicates<br>- Y2H plate assays: 3 biological replicates (one replicate is shown)<br>- Fluoresecence microscopy: 3 biological replicates (one replicate is shown)<br>- CoIP: 3 biological replicates (one replicate is shown)<br>- Seed germination: 3 biological replicates (60 seeds per replicate)<br>- RT-qPCR: 3 biological replicates<br>- EMSA: 3 biological replicates (one replicate is shown)<br>- Transactivation assays: 3 biological replicates<br>- Immunoblots: 3 biological replicates (one replicate is shown)<br>- ChIP-qPCR: 3 biological replicates<br>- Measurement of ABA content: 3 biological replicates |
| Data exclusions | No data were excluded. |
| Replication | All experiments were conducted at least three times (biological replicates). Statistical analyses are described in the figure legends and the source data file. |
| Randomization | Allocation of test plants was random. There was no targeted selection of individual plants for specific experiments or treatments. |
| Blinding | Blinding is uncommon in plant biology and was not used in the study. |

# Reporting for specific materials, systems and methods

We require information from authors about some types of materials, experimental systems and methods used in many studies. Here, indicate whether each material, system or method listed is relevant to your study. If you are not sure if a list item applies to your research, read the appropriate section before selecting a response.

## Materials & experimental systems

| n/a | Involved in the study | |
|---|---|---|
| ☐ | ☒ | Antibodies |
| ☒ | ☐ | Eukaryotic cell lines |
| ☒ | ☐ | Palaeontology and archaeology |
| ☒ | ☐ | Animals and other organisms |
| ☒ | ☐ | Human research participants |
| ☒ | ☐ | Clinical data |
| ☒ | ☐ | Dual use research of concern |

## Methods

| n/a | Involved in the study | |
|---|---|---|
| ☒ | ☐ | ChIP-seq |
| ☒ | ☐ | Flow cytometry |
| ☒ | ☐ | MRI-based neuroimaging |

## Antibodies

| | |
|---|---|
| Antibodies used | - anti-phyA: Agrisera, Vännäs, Sweden; Cat. no. AS07 220; polyclonal, rabbit, dilution 1:1,500<br>- anti-phyB: Hirschfeld, M., Tepperman, J. M., Clack, T., Quail, P. H. & Sharrock, R. A. Coordination of phytochrome levels in phyB mutants of Arabidopsis as revealed by apoprotein-specific monoclonal antibodies. Genetics 149, 523–535 (1998); monoclonal, mouse, B6-B3, dilution 1:250<br>- anti-HA: BioLegend, San Diego, CA, USA; Cat. no. 901502; monoclonal, mouse, 16B12, dilution 1:2,000<br>- anti-ACTIN: Sigma-Aldrich, St. Louis, MO, USA; Cat. no. A0480, monoclonal, mouse; 1:3,000 dilution<br>- Alkaline phosphatase goat anti-rabbit IgG: Vector Laboratories, Burlingame, CA, USA; Cat. no. AP-1000; dilution 1:7,500<br>- Alkaline phosphatase horse anti-mouse IgG: Vector Laboratories, Burlingame, CA, USA; Cat. no. AP-2000; dilution 1:10,000<br>- anti-GFP MicroBeads: Miltenyi Biotech; Cat. no. 130-091-125 |
| Validation | - anti-phyA: https://www.agrisera.com/en/artiklar/phytochrome-a.html<br>- anti-phyB: Hirschfeld, M., Tepperman, J. M., Clack, T., Quail, P. H. & Sharrock, R. A. Coordination of phytochrome levels in phyB |

mutants of Arabidopsis as revealed by apoprotein-specific monoclonal antibodies. Genetics 149, 523–535 (1998)
- anti-HA: https://www.biolegend.com/en-us/products/purified-anti-ha-11-epitope-tag-antibody-11374
- anti-ACTIN: https://www.sigmaaldrich.com/DE/de/product/sigma/a0480
