# Supplementary figures and images for: The phytochrome interacting proteins ERF55 and ERF58 repress light-induced seed germination in Arabidopsis thaliana
Source: Nat Commun. 2022 Mar 29;13:1656. doi: 10.1038/s41467-022-29315-3 (PMC8964797; doi:10.1038/s41467-022-29315-3)

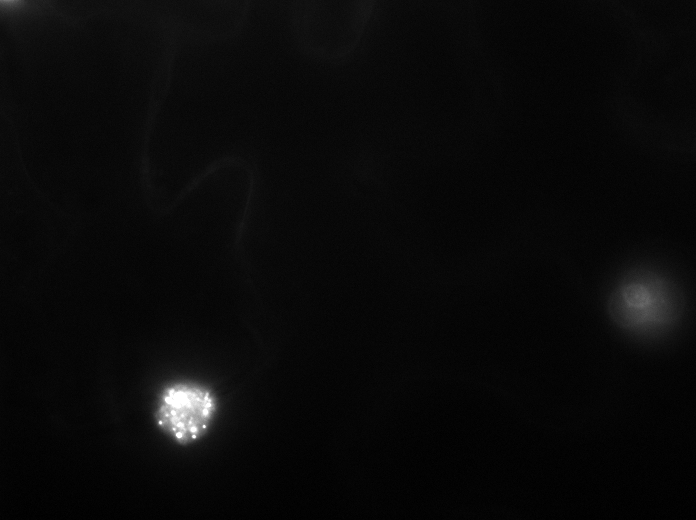

Supplement: Supplementary file 7 — Source Data [file 41467_2022_29315_MOESM7_ESM.zip › Source Data/Additional Source Data --- Fig 1c and Suppl Fig 1b --- Microscopy/Fig 1c_HA-YFP-ERF58+phyB-CFP_CFP.tif]

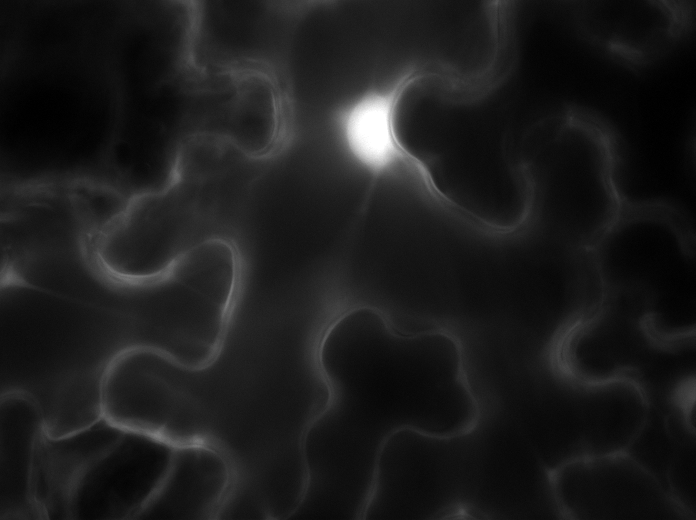

Supplement: Supplementary file 7 — Source Data [file 41467_2022_29315_MOESM7_ESM.zip › Source Data/Additional Source Data --- Fig 1c and Suppl Fig 1b --- Microscopy/Fig 1c and Fig S1b_YFP+CFP_CFP.tif]

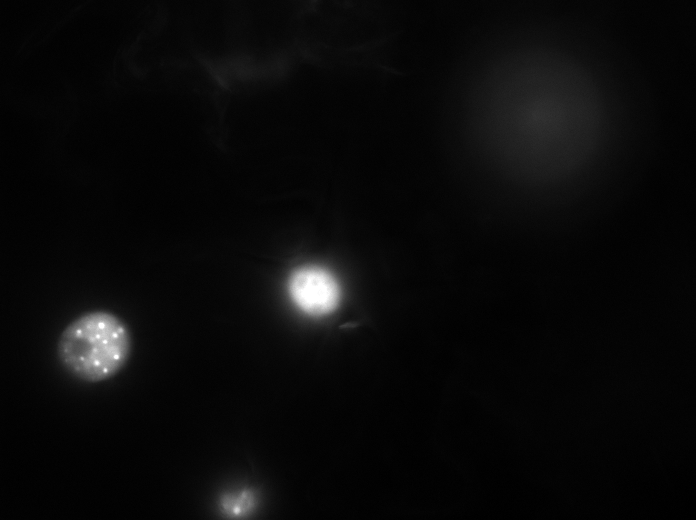

Supplement: Supplementary file 7 — Source Data [file 41467_2022_29315_MOESM7_ESM.zip › Source Data/Additional Source Data --- Fig 1c and Suppl Fig 1b --- Microscopy/Fig S1b_HA-YFP-ERF55+phyA-NLS-CFP_CFP.tif]

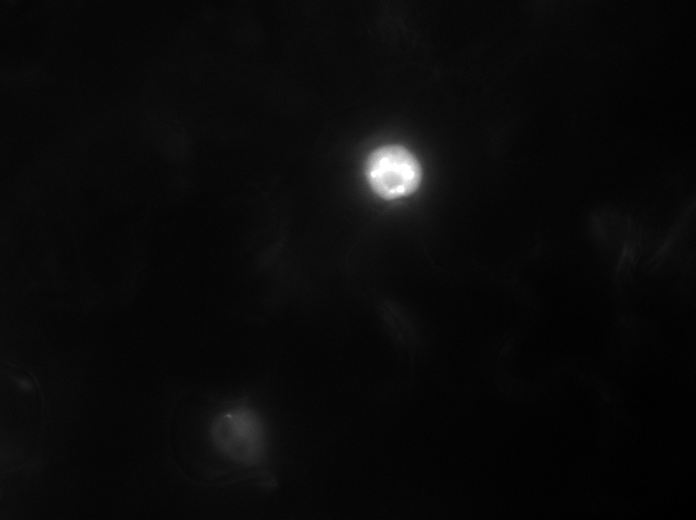

Supplement: Supplementary file 7 — Source Data [file 41467_2022_29315_MOESM7_ESM.zip › Source Data/Additional Source Data --- Fig 1c and Suppl Fig 1b --- Microscopy/Fig S1b_HA-YFP-ERF55+phyB-CFP_CFP.tif]

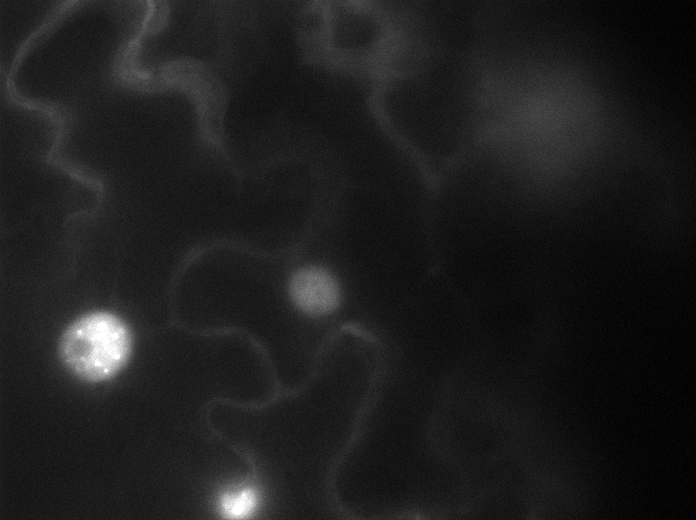

Supplement: Supplementary file 7 — Source Data [file 41467_2022_29315_MOESM7_ESM.zip › Source Data/Additional Source Data --- Fig 1c and Suppl Fig 1b --- Microscopy/Fig S1b_HA-YFP-ERF55+phyA-NLS-CFP_YFP.tif]

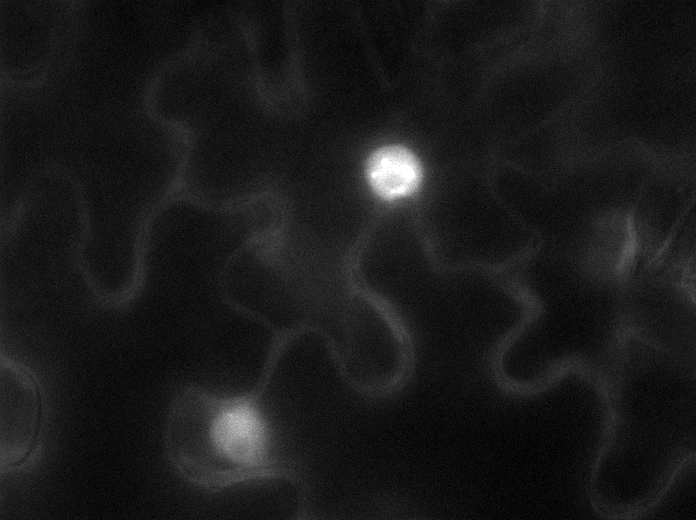

Supplement: Supplementary file 7 — Source Data [file 41467_2022_29315_MOESM7_ESM.zip › Source Data/Additional Source Data --- Fig 1c and Suppl Fig 1b --- Microscopy/Fig S1b_HA-YFP-ERF55+phyB-CFP_YFP.tif]

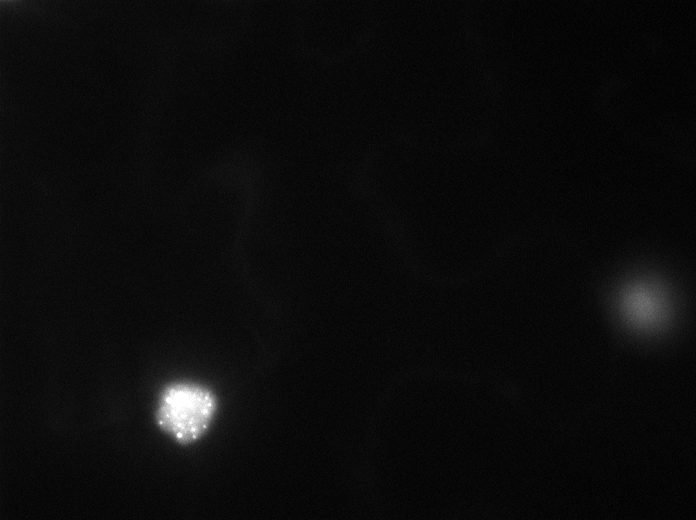

Supplement: Supplementary file 7 — Source Data [file 41467_2022_29315_MOESM7_ESM.zip › Source Data/Additional Source Data --- Fig 1c and Suppl Fig 1b --- Microscopy/Fig 1c_HA-YFP-ERF58+phyB-CFP_YFP.tif]

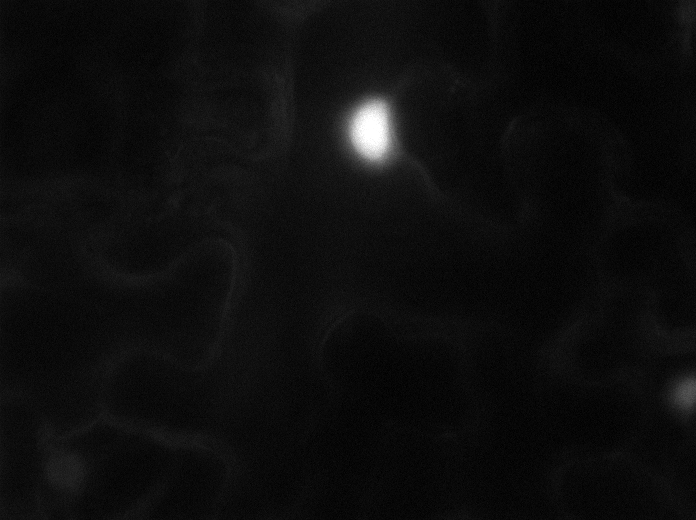

Supplement: Supplementary file 7 — Source Data [file 41467_2022_29315_MOESM7_ESM.zip › Source Data/Additional Source Data --- Fig 1c and Suppl Fig 1b --- Microscopy/Fig 1c and Fig S1b_YFP+CFP_YFP.tif]

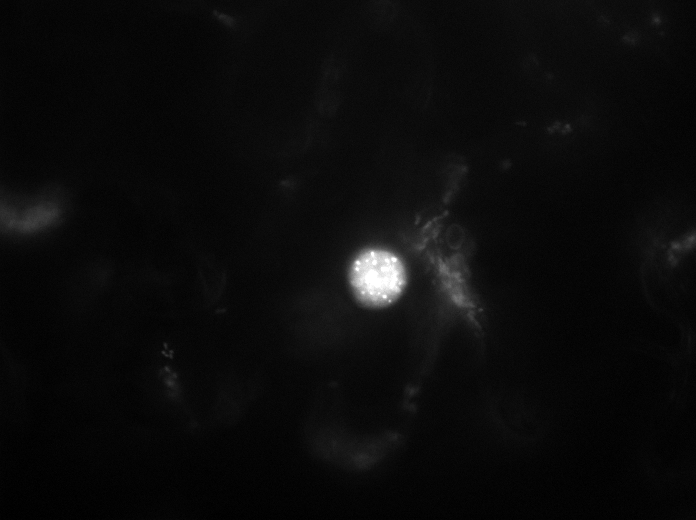

Supplement: Supplementary file 7 — Source Data [file 41467_2022_29315_MOESM7_ESM.zip › Source Data/Additional Source Data --- Fig 1c and Suppl Fig 1b --- Microscopy/Fig 1c_HA-YFP-ERF58+phyA-NLS-CFP_CFP.tif]

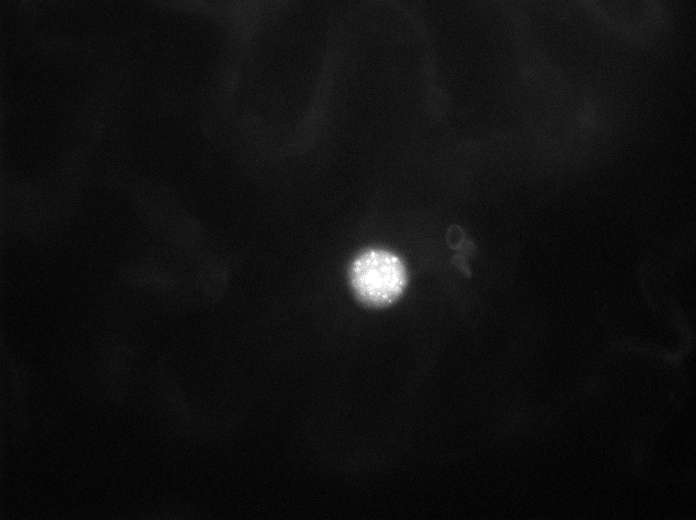

Supplement: Supplementary file 7 — Source Data [file 41467_2022_29315_MOESM7_ESM.zip › Source Data/Additional Source Data --- Fig 1c and Suppl Fig 1b --- Microscopy/Fig 1c_HA-YFP-ERF58+phyA-NLS-CFP_YFP.tif]
